# RSAD2 is abundant in atherosclerotic plaques and promotes interferon-induced CXCR3-chemokines in human smooth muscle cells

**DOI:** 10.1038/s41598-024-58592-9

**Published:** 2024-04-08

**Authors:** Assim Hayderi, Ashok K. Kumawat, Vladimir S. Shavva, Mats Dreifaldt, Birgitta Sigvant, Marcelo H. Petri, Björn Kragsterman, Peder S. Olofsson, Allan Sirsjö, Liza U. Ljungberg

**Affiliations:** 1 School of Medical Sciences, Örebro University, Örebro, Sweden; 2 Laboratory of Immunobiology, Division of Cardiovascular Medicine, Department of Medicine, Center for Bioelectronic Medicine, Solna, Karolinska Institutet, Karolinska University Hospital, Stockholm, Sweden; 3 Department of Cardiothoracic Surgery and Vascular Surgery, Örebro University Hospital, Örebro, Sweden; 4 Department of Surgical Sciences, Uppsala University, Uppsala, Sweden; 5 Department of Surgery, Västmanlands Hospital Västerås, Västerås, Sweden; 6 Institute of Bioelectronic Medicine, Feinstein Institutes for Medical Research, Manhasset, NY USA; 7 Centre for Clinical Research and Education, Region Värmland, Karlstad, Sweden

**Keywords:** Cell biology, Molecular biology, Molecular medicine

## Abstract

In atherosclerotic lesions, monocyte-derived macrophages are major source of interferon gamma (IFN-γ), a pleotropic cytokine known to regulate the expression of numerous genes, including the antiviral gene RSAD2. While RSAD2 was reported to be expressed in endothelial cells of human carotid lesions, its significance for the development of atherosclerosis remains utterly unknown. Here, we harnessed publicly available human carotid atherosclerotic data to explore RSAD2 in lesions and employed siRNA-mediated gene-knockdown to investigate its function in IFN-γ-stimulated human aortic smooth muscle cells (hAoSMCs). Silencing RSAD2 in IFN-γ-stimulated hAoSMCs resulted in reduced expression and secretion of key CXCR3-chemokines, CXCL9, CXCL10, and CXCL11. Conditioned medium from RSAD2-deficient hAoSMCs exhibited diminished monocyte attraction in vitro compared to conditioned medium from control cells. Furthermore, RSAD2 transcript was elevated in carotid lesions where it was expressed by several different cell types, including endothelial cells, macrophages and smooth muscle cells. Interestingly, RSAD2 displayed significant correlations with CXCL10 (*r* =  0.45, *p* = 0.010) and CXCL11 (*r* = 0.53, *p* = 0.002) in human carotid lesions. Combining our findings, we uncover a novel role for RSAD2 in hAoSMCs, which could potentially contribute to monocyte recruitment in the context of atherosclerosis.

## Introduction

Virus inhibitory protein endoplasm reticulum-associated interferon inducible (VIPERIN) also known as radical S-adenosyl-L-methionine domain containing 2 (RSAD2) is an antiviral gene that has garnered increasing attention in recent years^1^. Since its discovery two decades ago, RSAD2 has been extensively studied for its role against viral infections and is now a well-established antiviral protein with many known mechanisms against viral infections^2–5^. Emerging evidence suggests that RSAD2 may play a role in atherosclerosis, as it interferes with cellular processes that are central in the development and progression of atherosclerosis^6,7^. For instance, RSAD2 has recently been reported to interact with key proteins involved in lipid metabolism and transient expression of RSAD2 in human embryonic kidney cells has been shown to inhibit cholesterol biosynthesis^6^. Intrinsic expression of RSAD2 in brown adipose tissues is linked to reduced fatty acid β-oxidation and thermogenesis in mice^7^. In addition, RSAD2 has been shown to be crucial for the maturation of dendritic cells and may also participate in differentiation of macrophages and T cells^8–10^. Altered lipid metabolism and dysregulated immune response are key drivers of atherosclerosis^11,12^.

Although RSAD2 has been reported to be basally expressed in liver, heart and adipose tissues of mice, most tissues and cell do not have any basal expression of RSAD2^7^. These cells, however, express RSAD2 upon direct stimulation with interferons or upon encounter with infectious agents, which may directly or indirectly induce RSAD2 by inducing type I interferons^13,14^. In addition to type I interferons, both type III interferons and interferon gamma (IFN-γ), a major pro-atherogenic cytokine, have been reported to induce RSAD2 in various cell types, including endothelial cells and coronary smooth muscle cells^13,15^. More importantly, RSAD2 has been shown to be expressed in human carotid plaque endothelial cells^15^. However, its relevance for the development and progression of atherosclerosis and its function in vascular cells remain utterly unknown. Considering the involvement of RSAD2 in regulation of lipid metabolism and its participation in maturation and differentiation of immune cells, two central processes in atherosclerosis^11,12^, we aimed to explore RSAD2 in human carotid atherosclerotic tissues and elucidate its function in human aortic smooth muscle cells (hAoSMCs).

## Results

### RSAD2 is expressed in plaque macrophages, endothelial and smooth muscle cells

**Figure 1:**
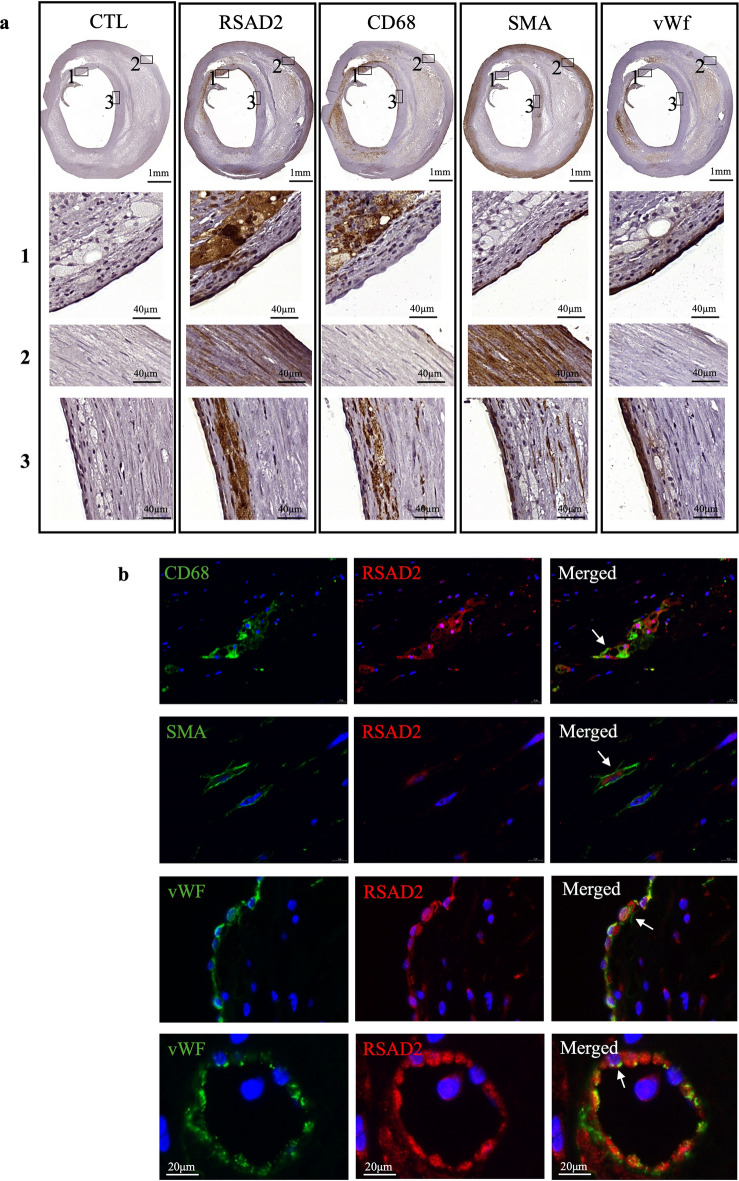
RSAD2 is expressed in human carotid atherosclerotic lesions and co-localize with vascular cells and macrophages. (**a**) Immunohistochemical staining of human carotid atherosclerotic lesions with control, RSAD2, CD68, SMA and vWF antibodies. The images in the three lower panels labeled with 1, 2 and 3 are magnification of the areas within the boxes in the uppermost images labeled with 1, 2 and 3. SMA, smooth muscle actin; vWF, Von Willebrand factor. (**b**) Immunofluorescence staining of human carotid lesions stained for RSAD2 in red and different cell markers, CD68, SMA and vWF in green. SMA, smooth muscle actin; vWF, Von Willebrand factor; CD68, cluster of differentiation 68; RSAD2, radical S-adenosyl-L-methionine domaine containing 2.

RSAD2 was reported to be expressed in carotid plaque endothelial cells^15^. To confirm this and examine whether its expression is restricted to plaque endothelial cells, we conducted single and double staining of human carotid plaques. Immunohistochemical analysis revealed high expression of RSAD2 in regions that were abundant in macrophages (CD68) and SMCs (smooth muscle actin alpha, SMA) (Fig. 1a and Supplementary Fig. S1). Moreover, RSAD2 was found to be expressed in areas with endothelial cells expressing von Willebrand factor (vWF) (Fig. 1a). Immunofluorescence co-staining for RSAD2 in combination with CD68, SMA and vWF revealed RSAD2 expression in macrophages, endothelial cells, and SMCs (Fig. 1b). In addition, we found that both carotid artery luminal endothelial cells (Fig. 1b) and intimal microvascular endothelial cells expressed RSAD2 (Fig. 1b).

### IFN-γ strongly and persistently induces RSAD2 in hAoSMCs

**Figure 2:**
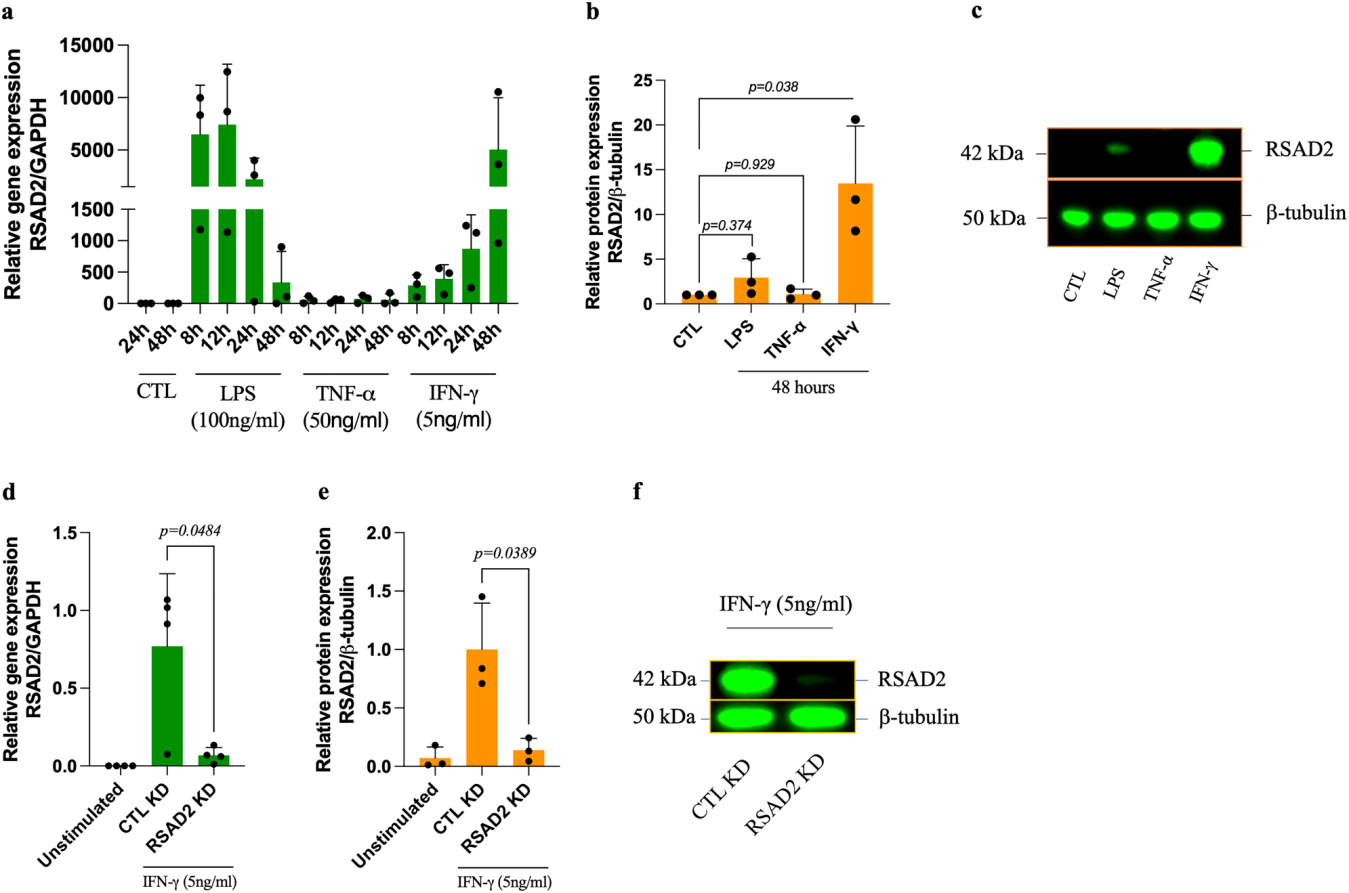
Inflammatory mediators induce RSAD2 in human aortic smooth muscle cells and RSAD2-siRNA efficiently reduces IFN-γ-induced RSAD2 expression. (**a**) Relative mRNA expression of RSAD2 in hAoSMCs stimulated with IFN-γ (5ng/ml), LPS (100µg/ml) or TNF-α (50ng/ml) for 8–48 h (n = 3). (**b**) Relative protein expression of RSAD2 in hAoSMCs exposed to LPS (100µg/ml), TNF-α (50ng/ml) or IFN-γ (5ng/ml) for 48 h (n = 3). (**c**) Representative cropped Western blot image of RSAD2 and β-tubulin from hAoSMCs exposed to LPS, TNF-α or IFN-γ for 48 h (full-length Western Blot images are shown in supplementary fig. S2a). (**d**) Relative mRNA (n = 4) and (**e**) protein (n = 3) expression of RSAD2 in hAoSMCs transfected with 30pmol of RSAD2-targeting or scramble siRNAs and stimulated with IFN-γ for 44 h (normalized to the average of CTL KD). (**f**) Representative cropped Western Blot image of RSAD2 and β-tubulin from IFN-γ-stimulated hAoSMCs transfected with RSAD2-targeting or scramble siRNAs (full-length Western Blot images are shown in Supplementary Fig. S3a). The data are presented as mean ± SD. One-way ANOVA followed by Bonferroni's multiple comparison test was used to evaluate statistical significance. *p* value < 0.05 is considered statistically significant. LPS, lipopolysaccharide; TNF-α, tumor necrosis factor alpha; IFN-γ, interferon gamma; RSAD2, radical S-adenosyl-L-methionine domaine containing 2.

Due to high abundance of SMCs in human carotid atherosclerotic lesions and the fact that these cells express RSAD2 in human carotid lesions, we sought to further study the function of RSAD2 in cultured hAoSMCs. These cells, however, do not express RSAD2 basally, but RSAD2 has been shown to be induced by pathogen-associated molecular patterns (PAMPs) and cytokines in several different cell types^8,13,15^. To select a potent inducer of RSAD2 for hAoSMCs, we stimulated these cells with IFN-γ (5 ng/ml), LPS (100 ng/ml) and TNF-α (50 ng/ml) for different timepoints ranging from 8 to 48 h and assessed RSAD2 mRNA levels by qRT-PCR. We observed a strong, but not statistically significant, induction of RSAD2 mRNA by LPS and IFN-γ, whereas TNF-α caused only a minor induction (< 166-fold change) (Fig. 2a).

To evaluate the protein expression of RSAD2, we analyzed lysate from the hAoSMCs treated with IFN-γ, LPS or TNF-α for 48 h utilizing Western blot. While the protein expression of RSAD2 was strongly and significantly elevated in IFN-γ-stimulated cell lysate (*p* = 0.038) (Fig. 2b, c), LPS treated cells displayed a slight and non-statistically significant induction. Lysate from TNF-α-stimulated cells did not show any RSAD2 protein 48 h post stimulation. A representative Western blot image is shown in Fig. 2c, and the full-length Western blot images are presented in Supplementary Fig. S2a (lane 2–5).

### siRNA-mediated knockdown of RSAD2 downregulates the expression of CXCR3 ligands in hAoSMCs

As IFN-γ was a potent inducer of RSAD2 in hAoSMCs, we used IFN-γ to induce RSAD2 in our subsequent experiments. To explore RSAD2-related functions in hAoSMCs, we transfected hAoSMCs with RSAD2 siRNA prior to stimulation with IFN-γ. The knockdown efficiency was evaluated by qRT-PCR and Western blot. Our results demonstrate a 91% reduction of the RSAD2 mRNA and 86% reduction of RSAD2 protein in IFN-γ-stimulated hAoSMCs transfected with RSAD2-targeting siRNA (Fig. 2d, e). A representative Western blot image of the knockdown efficiency is shown in Fig. 2f, and the full-length Western blot images are presented in Supplementary Fig. S3a (lane 4 and 5).

**Figure 3:**
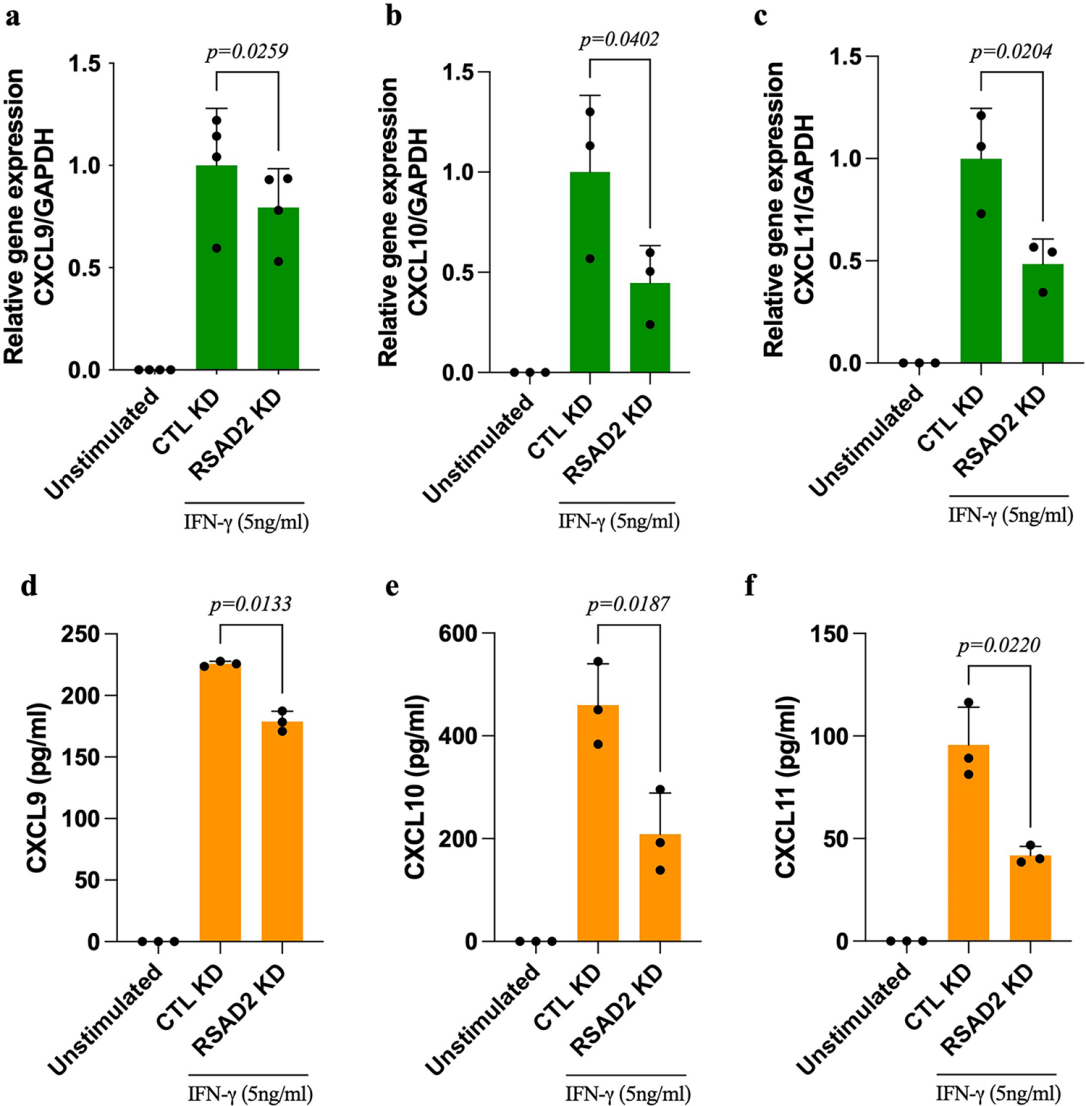
Knockdown of RSAD2 in IFN-γ-stimulated human aortic smooth muscle cells results in reduced synthesis and secretion of CXCR3 ligands. Relative mRNA expression of (**a**) CXCL9 (n = 4), (**b**) CXCL10 (n = 3) and (**c**) CXCL11 (n = 3) in hAoSMCs transfected with RSAD2-targeting or scramble siRNAs and treated with IFN-γ for 44 h (normalized to the average of CTL KD). Protein quantification of (**d**) CXCL9 (n = 3), (**e**) CXCL10 (n = 3) and (**f**) CXCL11 (n = 3) in supernatant of hAoSMCs transfected with RSAD2-targeting or scramble siRNAs and stimulated with IFN-γ for 44 h. One-way ANOVA followed by Bonferroni’s multiple comparison test was employed to test for statistical significance. *p* value < 0.05 is considered statistically significant. CXCL9/10/11, CXC-motif ligand 9/10/11.

To explore whether silencing of RSAD2 affects the release of inflammatory mediators, supernatants from IFN-γ-stimulated hAoSMCs (n = 1), transfected with RSAD2 or scramble siRNA, were screened using OLINK proteomics’ inflammation panel. Out of the 92 proteins in this panel, 53 were detected in our cell supernatants. Among these, the three CXCR3 ligands, CXCL9, CXCL10 and CXCL11, as well as CCL3, were found to be reduced in RSAD2-siRNA transfected cells (Supplementary Fig. S4). To validate these observations, we quantified the mRNA and protein expression of these chemokines using qRT-PCR and ELISA. While CCL3 was not detected by qRT-PCR (CT-values > 35), we confirmed a significant reduction in the levels of CXCL9 (*p* = 0.026 and 0.013), CXCL10 (*p* = 0.035 and 0.019) and CXCL11 (*p* = 0.020 and 0.022) on transcriptional and protein levels, respectively, in RSAD2 siRNA-transfected hAoSMCs (Fig. 3a–f).

### Supernatant from RSAD2 siRNA-transfected cells attracts fewer monocytes in vitro

**Figure 4:**
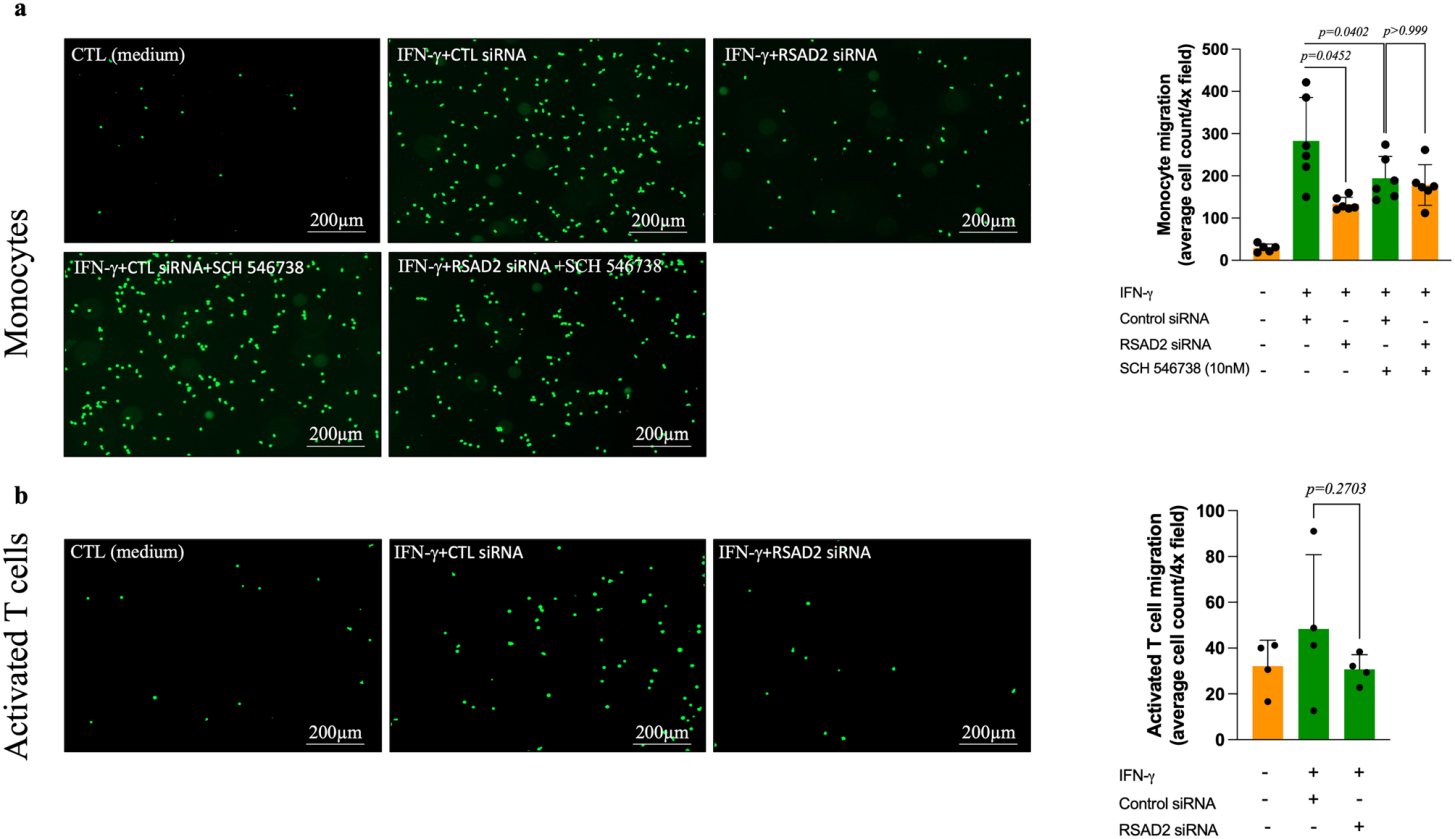
Supernatant from RSAD2 knockdown cells attracts fewer monocytes in vitro. Representative images illustrating migrated human (**a**) monocyte (n = 6) and (**b**) activated T cells (n = 4) towards conditioned medium from IFN-γ-stimulated hAoSMCs that were transfected with RSAD2-targeting or scramble siRNA for 44 h. Monocytes were additionally incubated with 10nM of SCH 546,738, a selective CXCR3 antagonist, to evaluate whether the observed effect is mediated by CXCR3 ligands. Average cells count per 4 × field in the lower chamber of the trans-wells after migration of the monocytes and activated CD3^+^ T cells are shown in the bar graphs. One-way ANOVA followed by Bonferroni’s multiple comparison test was used to assess statistical significance. *p* value < 0.05 is considered statistically significant. IFN-γ, interferon gamma; RSAD2, radical S-adenosyl-L-methionine domaine containing 2; CD3, cluster of differentiation 3.

CXCL9, CXCL10 and CXCL11 mediate the recruitment of CXCR3-expressing cells, which include activated T cells and monocytes. Examination of the CXCR3 transcripts in cells of human carotid atherosclerotic lesions^16^ indicates T cells as the major cell type expressing CXCR3 although CXCR3 transcript was also found in cell clusters representing macrophages, endothelial and smooth muscle cells. To test whether a reduction in the levels of these chemokines, caused by RSAD2 knockdown, also affects the migration of T cells and monocytes in vitro, we evaluated the migratory response of CD14^+^ monocytes and activated CD3^+^ T cells towards conditioned medium from hAoSMCs transfected with scramble or RSAD2-targeting siRNA using Boyden chambers. Recombinant human CXCL12, and the combination of CXCL11 and CXCL12 were used as positive controls for monocytes and T cells, respectively (Supplementary Fig. S5a,b). We observed a significant reduction in the number of monocytes (*p* = 0.045) migrating towards conditioned medium from IFN-γ-treated hAoSMCs that were transfected with RSAD2-targeting siRNA as compared to supernatant from scramble siRNA-transfected cells (Fig. 4a). Preincubation of the monocytes with 10 nM SCH 546738, a selective CXCR3 antagonist, for 5 min prior to migration caused a significant reduction in the number of migrated monocytes towards conditioned medium from scramble siRNA-transfected hAoSMCs, suggesting that the observed effect is mediated by CXCR3 ligands (Fig. 4a). Activated T cells showed a reduced tendency of migration towards conditioned medium from cells transfected with RSAD2 siRNA. This was however not statistically significant (Fig. 4b).

### RSAD2 mRNA is upregulated and correlates with CXCL10 and CXCL11 in human carotid plaque dataset

**Figure 5:**
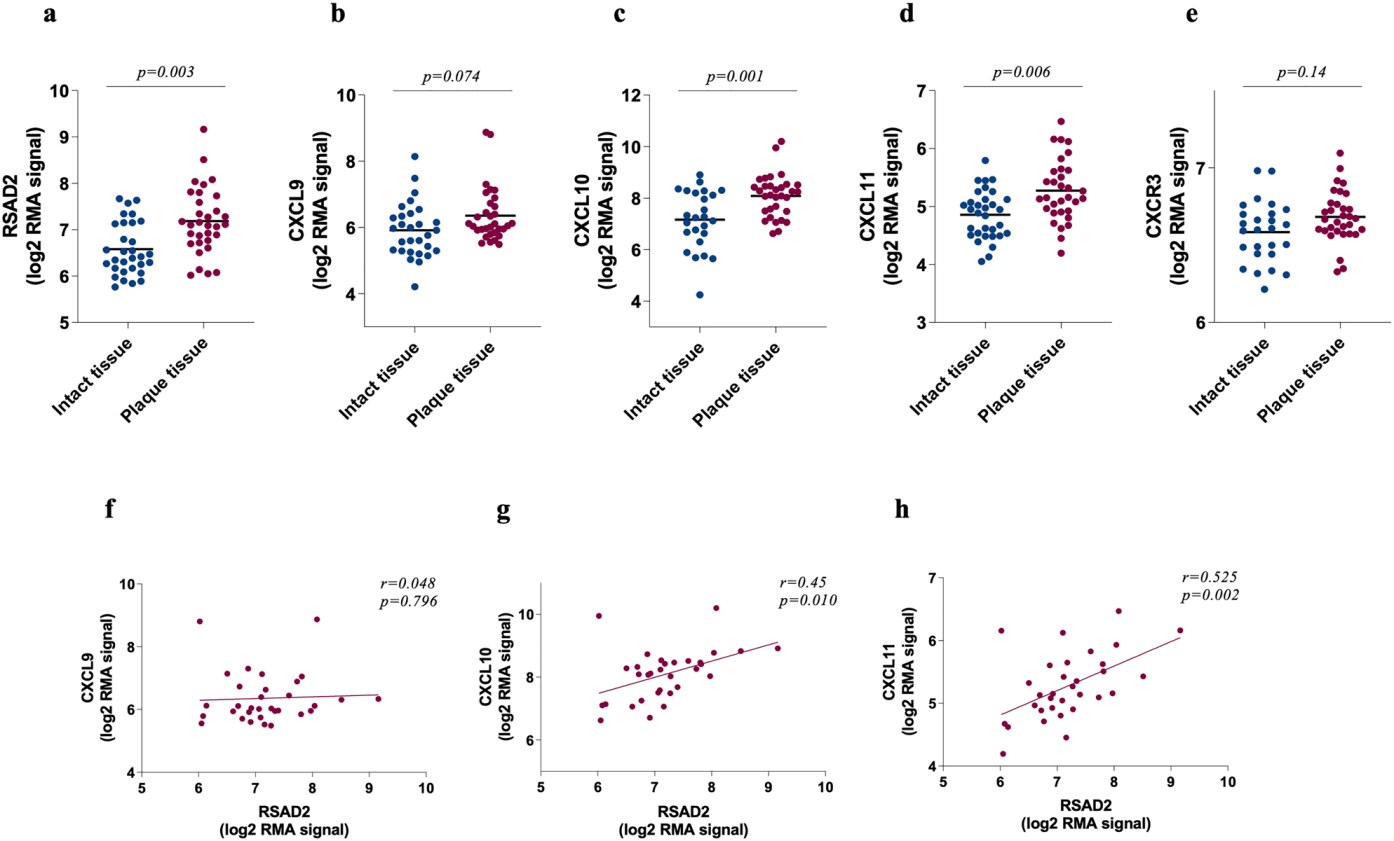
Transcripts of RSAD2, CXCR3 and its ligands are more abundant in human carotid lesions where RSAD2 mRNA also correlates with CXCL10 and CXCL11 mRNA. The transcripts of (**a**) RSAD2, (**b**) CXCL9, (**c**) CXCL10, (**d**) CXCL11 and (**e**) CXCR3 in human atherosclerotic lesions (red circles) and adjacent intact tissues (blue circles) of matched patients with hypertension (n = 32) (GEO accession: GSE43292). Paired t-test followed by Benjamini and Hochberg corrections were employed to obtain adjusted *p* values and Pearson’s correlation was used to assess the relationship between RSAD2 mRNA and (**f**) CXCL9, (**g**) CXCL10 and (**h**) CXCL11 mRNA in human carotid lesions. Adjusted *p* values and Pearson’s correlation coefficients are indicated in the figures. *p* value < 0.05 is considered statistically significant. CXCR3, CXC chemokine receptor 3; CXCL9/10/11, CXC-motif chemokine 9/10/11; RSAD2, radical S-adenosyl-L-methionine domaine containing 2.

Using the biobank collection of plaque samples from hypertensive patients undergoing carotid endarterectomy at Lyon University Hospital (GEO accession: GSE43292), we examined the total mRNA levels of RSAD2, CXCR3 and its ligands in atheroma plaques and adjacent macroscopically intact tissues of the same patients. The expression of RSAD2 (*p* = 0.003), CXCL10 (*p* = 0.001) and CXCL11 (*p* = 0.006) were found to be significantly higher in atheroma plaques (plaque tissue) as compared to adjacent macroscopically intact tissues (intact tissue) (Fig. 5a,c,d). The expression of CXCL9 and CXCR3 however were not statistically different between lesions and adjacent intact tissues (Fig. 5b,e). Moreover, RSAD2 transcript displayed a significant correlation with CXCL10 (*r* = 0.45, *p* = 0.01) and CXCL11 (*r* = 0.53, *p* = 0.002) but not CXCL9 (*r* = 0.05, *p* = 0.80) in plaque tissues (Fig. 5f–h). We could not see any correlation between RSAD2 and CXCR3 although CXCR3 correlated with CXCL9, CXCL10 and CXCL11 (Supplementary Fig. S6a–d).

## Discussion

Here, we report that RSAD2 is expressed in various cell types within human carotid atherosclerotic plaques, including endothelial cells, smooth muscle cells and macrophages. RSAD2 deficiency in IFN-γ-stimulated hAoSMCs results in significant reduction in the expression and secretion of CXCL9, CXCL10 and CXCL11. Consequently, supernatant from RSAD2 deficient IFN-γ-stimulated hAoSMCs attracts fewer monocytes in vitro. Moreover, our analysis of microarray-based data from human carotid plaques indicates that RSAD2 mRNA is substantially higher in the plaque core in comparison to macroscopically intact proximal adjacent tissues of matched patients. Notably, within these plaques, RSAD2 displays a significant positive correlation with both CXCL10 and CXCL11.

Previously, RSAD2 expression had been attributed solely to plaque endothelial cells^15^. However, our findings reveal, for the first time, that RSAD2 is also expressed by SMCs and macrophages within carotid atherosclerotic plaques, although its significance for the development of atherosclerosis has remained completely unknown. Our analysis of commercially obtained healthy hAoSMCs revealed no inherent expression of RSAD2, indicating that RSAD expression in SMCs within atherosclerotic lesions may be induced by inflammatory stimuli. Notably, viral or bacterial components, as well as interferons—each of which has been detected within atherosclerotic lesions—have previously been reported to induce RSAD2 expression in various cell types, including SMCs and human umbilical vein endothelial cells^7,8,13,15^. Even more surprisingly, it has been reported that hypertension is associated with elevated RSAD2 expression in peripheral blood mononuclear cells of systemic sclerosis patients^17^. These finding not only confirm the prior observations by Olofsson and colleagues, who reported RSAD2 expression in plaque endothelial cells, but also expand upon their findings by revealing RSAD2 expression in macrophages and SMCs, the two major cell types within atherosclerotic lesions.

SMCs account for at least 50% of foam cells within lesions and secrete hefty amounts of chemokines, which is crucial for immune cell recruitment during atherosclerosis^18,19^. Silencing RSAD2 in IFN-γ-stimulated hAoSMCs resulted in decreased expression of CXCL9, CXCL10 and CXCL11. RSAD2 has previously been reported to regulate chondrogenic differentiation by targeting the expression and secretion of CXCL10^20^. These chemokines are proatherogenic and known to mediate the recruitment of CXCR3 expressing T cells and monocytes^21^. Pharmacological blockade or targeted deletion of CXCR3 in apoE−/− has been reported to result in diminished plaque size. This effect is accompanied by a reduced influx of Th1 cells and increased migration of regulatory T cells^22^. In line with this findings, CXCL10 deficient apoE−/− mice exhibit reduced atherogenesis, followed by elevated numbers of heightened activity of regulatory T cells^23^. Given the importance of these chemokines for the recruitment of CXCR3^+^ leukocytes, we evaluated the migratory response of activated CD3^+^ T cells and primary monocytes towards conditioned medium from RSAD2 deficient cells in vitro. While we did not observe any difference in the migratory response of activated CD3^+^ T cells, we observed a significant reduction in the number of monocytes migrating towards conditioned medium from RSAD2 deficient hAoSMCs and this was indeed mediated by CXCL9, CXCL10, and CXCL11, as assessed by inhibition of CXCR3 on monocytes by a selective inhibitor. This is in line with a previous study where the authors reported monocytes but not T cells migrating towards CXCL9, CXCL10 and CXCL11 in vitro^24^. These findings suggest that IFN-γ-induced CXCL9, CXCL10 and CXCL11 may, at least partially, be regulated by RSAD2 in IFN-γ-stimulated hAoSMCs and that this may be important for the recruitment of monocytes. During screening for potential target genes with OLINK technology, we also found CCL3 to be reduced in the supernatant of RSAD2 deficient hAoSMCs. However, the levels were very low and mRNA for CCL3 could not be detected, neither in treated nor in untreated cells.

In our analysis of human atherosclerotic microarray data, we investigated the presence of RSAD2 within lesions and its relationship with CXCR3 and its ligands. We found RSAD2, CXCL10 and CXCL11, but not CXCL9 and CXCR3, to be significantly upregulated in lesions compared to adjacent intact tissues. When examining the association between RSAD2 and CXCR3 with its ligands, we observed a positive correlation between RSAD2, CXCL10 and CXCL11, while there was no correlation between RSAD2 and CXCR3. More interestingly, when we looked at the top 20 genes with the highest correlation coefficients towards RSAD2, we found the majority to be interferon-stimulated genes (Supplementary Table 1). Although the expression of these interferon-induced genes was higher in plaque core, the interferon levels did not differ between plaque core and adjacent intact tissues. This may suggest a significantly higher interferon activity despite low levels of interferon transcripts in lesions. Quantification of IFN-γ in atherosclerotic lesions, by Frostegård and colleagues, revealed detectable IFN-γ protein in only 30% of human carotid lesions^25^. Hence, quantifying interferon stimulated genes as an indicator of interferon activity may be more accurate although interferon stimulated genes, such as RSAD2, may be weakly induced, at least on mRNA level, by other cytokines, such as TNF-α.

In the current study, we report that the antiviral gene RSAD2 is expressed in a wide range of cells within human carotid atherosclerotic lesions. Silencing RSAD2 in IFN-γ-stimulated hAoSMCs results in reduced expression and secretion of the CXCL9, CXCL10 and CXCL11, and supernatant from these cells is less attractive for monocytes in vitro. Whether the regulation of these chemokines by RSAD2 holds true for other cell types found within lesion has not been evaluated here, which we acknowledge is a limitation of this study. It would therefore be interesting to evaluate the function of RSAD2 and assess its link with these chemokines in other cell types, including macrophages, T cells and dendritic cells as these cells within lesions were positive for RSAD2 and RSAD2 has been reported to have some role in the maturation and polarization of these cells^8–10,16^. Another limitation of our study is not being able to evaluate the consequence of overexpression of RSAD2 in hAoSMCs, since empty plasmid itself induced RSAD2 in these cells, which poses a challenge in finding a suitable control.

## Methods

### Culturing and treatment of hAoSMCs

hAoSMCs (catnr: C-007-5C, lot: 2164581, Life Technologies, Carlsbad, California, USA and Cell Applications Inc. San Diego, California, USA) were cultured in Medium 231 (Life Technologies) supplemented with smooth muscle growth supplement (complete SMC medium) (Life Technologies) and 10 U/ml penicillin and streptomycin (PEST) (Life Technologies) at 37 °C and 5% CO_2_. The medium was changed every 48 h and the cells up to passage 6 were sub-cultured or used for experiments upon 80–90% confluency by detachment with trypsin (Life Technologies). hAoSMCs were seeded at a density of 1.25 × 10^5^ cells/well in 6-well culture plates (Sarstedt Inc., Nümbrecht, Germany) overnight and subjected to different treatments for various timepoints on the following day. Cells and supernatants were stored at − 80 °C until further analyses.

### siRNA-mediated gene silencing in hAoSMCs

hAoSMCs were seeded at a density of 1.25 × 10^5^ cells/well in 6-well plates overnight. The cells were rinsed with 1 ml Opti-Mem (Life Technologies) and incubated with 30 pmol of Stealth RNAi Negative Control (Life Technologies) or a mixture of three Stealth siRNA targeting RSAD2 mRNA (10 pmol of each) (Thermo Scientific, Waltham, Massachusetts, USA) in 4 μl Lipofectamin2000 (Life Technologies) dissolved in Opti-Mem for 4 h. Fresh medium was added, and the cells were treated with 5 ng/ml IFN-γ (R&D systems, Minneapolis, Minnesota, USA) and incubated for additional 44 h at 37 °C and 5% CO_2_.

### OLINK proteomics

Supernatant from treated hAoSMCs (n = 1) was analyzed using OLINK proteomics’ inflammation panel (OLINK Bioscience AB, Uppsala, Sweden). OLINK utilizes proximity extension assay where two antibodies, tagged with complementary nucleotide sequences, are used to bind different epitopes of the same protein. Upon binding of these antibodies to their epitopes, the complementary nucleotide sequences hybridize and is amplified by PCR technique. The amplified PCR product is proportional to the amount of the protein and is reported as normalized protein expression on a log2 scale, which was used to calculate log2 fold change (FC).

### Quantitative real-time polymerase chain reaction (qRT-PCR)

Total RNA was isolated from frozen hAoSMCs using E.Z.N.A total RNA kit (Omega Bio-tek inc, Norcross, Giorgia, USA) as per supplier’s protocol. The quantity and purity of the total RNA were determined using NanoDrop 2000 Spectrophotometer (Thermo Scientific). Complementary DNA was reversely transcribed using 1 μg of total RNA with High-capacity cDNA reverse transcription kit (Thermo Scientific) in a Biometra UNO-Thermoblock (Biometra Biomedizinische Analytik Gmbh, Göttingen, Germany). qRT-PCR was conducted using LuminoCt® ReadyMix (Sigma-Aldrich, Saint Louis, Missouri, USA) to evaluate the expression of RSAD2 (Hs00369813_m1), CXCL9 (Hs00171065_m1), CXCL10 (Hs01124252_g1) and CXCL11 (Hs00171138_m1) (all primer-probes were purchased from Thermo Scientific) using QuantStudio 7 Flex Real-Time PCR System (Thermo Scientific). Gene expression was normalized to the expression of GAPDH (Hs99999905_m1). The relative expression of each gene was determined using a 6-point standard curve generated from serial dilutions of pooled cDNA. Data for the target genes (RSAD2, CXCL9, CXCL10 and CXCL11) were normalised to GAPDH. The average for CTL or CTL KD groups from 3-4 experiments was determined, and normalised data for all treatment groups, including the CTL and CTL KD, was normalised to this average prior to calculating the fold-change.

### Western blot

Frozen cells were lysed with radio-immunoprecipitation assay (RIPA) buffer (Millipore, Burlington, Massachusetts, USA) containing 1% protease inhibitor (Thermo Scientific). A small fraction of the cell lysate was used to determine total protein amount in each sample with Micro Bicinchoninic acid (BCA) Protein Assay Kit (Thermo Scientific) according to the supplier’s instructions. 40 μl of the remaining lysate was mixed with 10 μl of 5 × sodium dodecyl sulfate (SDS) and heated for 5 min at 95 °C. A total of 10 μg protein from each sample was loaded on to 4–12% NuPage Novex Bis–Tris gels (Thermo Scientific). Novex Sharp Pre-Stained Protein Standard (Thermo Scientific) and Magic Marker (Thermo Scientific) were prepared according to the manufacturer’s instructions and used as standard to determine the molecular size of proteins. The proteins were separated using 3-(N-morpholino) propanesulfonic acid (MOPS) running buffer (Life Technologies) at 140 V for 95 min. The proteins were then transferred to activated Immobilon-FL PVDF membrane (Millipore) at 125mA for 95 min. The membrane was blocked with 5% protease-free Albumin Fraction V (Carl Roth GmbH & Co. Kg, Karlsruhe, Germany) dissolved in TBS-T (0.01% Teween-20) for 1 h at RT and incubated with rabbit anti-human RSAD2 antibodies (1:1000, catnr. HPA041160, lot. A96738, Atlas antibodies, Stockholm, Sweden) overnight at + 4 °C. This antibody has been validated by Atlas Antibodies using RSAD2-transfected HEK293T cell lysate. After a thorough wash, the membrane was incubated with HRP-conjugated goat anti-rabbit IgGs (1:2000, catnr. 7074S, lot. 29, Cell Signaling Technology, Danvers, Massachusetts, USA) for 45 min at RT. The membrane was washed and incubated with Immobilon Chemiluminescent HRP substrate (Millipore) for 30 s prior to measuring the signal by ODYSSEY FC imaging system (LI-COR Biosciences, Lincoln, Nebraska, USA). The membranes were re-probed with mouse anti-human β-tubulin clone AA2 antibodies^26^ (1:2000, catnr. 05–661, lot. 2,913,549, Millipore) for 2 h at RT. After a thorough wash, the membrane was incubated with HRP-linked horse anti-mouse IgGs (1:2000, catnr. 7076S, lot. 33, Cell Signaling Technology) for 45 min at RT. Finally, the membrane was incubated with HRP substrate for 30 s and the signal intensity was measured. The images were analyzed with Image Studio (LI-COR). Data for RSAD2 was normalised to beta tubulin. The average for CTL or CTL KD groups from 3 experiments was determined, and the normalised data for all treatment groups, including CTL and CTL KD, was normalised to this average prior to calculating the fold-change.

### Enzyme-linked immunosorbent assay (ELISA)

ELISA was conducted in accordance with supplier’s protocols to quantify human CXCL9 (R&D systems), CXCL10 (ImmunoTools GmbH, Friesoythe, Germany) and CXCL11 (R&D systems) from supernatants of hAoSMCs subjected to scramble or RSAD2 targeting siRNA and IFN-γ. The absorbance was measured at 450 nm with Cytation 3 (BioTek, Winooski, Vermont, USA).

### Migration assay

Human blood from healthy volunteers was used to study migration of leukocytes in vitro. Fresh blood was collected from healthy donors in EDTA-coated tubes at Örebro University Hospital. The blood was diluted with equal volume of PBS, layered carefully on 10 ml of lymphoprep (STEMCELL Technologies, Vancouver, Canada) and centrifuged at 400×*g* and RT for 30 min without brake. The mid layer consisting of peripheral blood mononuclear cells (PBMCs) were transferred to a 50 ml falcon tube and washed twice with cold PBS containing 0.5 IU/ml heparin (LEO Pharma AB, Malmö, Sweden) at 400×*g* and 220×*g* for 10 respectively 6 min. The cell concentration was adjusted to 20 million cells/ml in PBS supplemented with 2% heat-inactivated FBS (Life Technologies) and 1% EDTA (Life Technologies). EasySep™ Human T cells enrichment and Human Monocyte isolation kits (STEMCELL Technologies) were used to isolate untouched CD3^+^ T cells and untouched CD14^+^ monocytes according to the supplier’s protocol. CD14^+^ monocytes were stained with BCECF-AM (Sigma-Aldrich) for 1 h at + 4 °C and washed twice with PBS containing 2% heat-inactivated FBS (Life Technologies) and 1% EDTA (Life Technologies) before adjusting the concentration to 250,000 cells/ml in complete SMC medium (Life Technologies). Stained CD14^+^ cells were incubated in the absence or presence of 10 nM of the selective CXCR3-antagonist, SCH 546,738 (MedChemExpress, New Jersey, USA) for 5 min and the cells (5 × 10^4^ cells in 200 μl) were transferred to the upper chambers of a pre-moistened 24-well transwell system with 5 μm pore size (SABEU GmbH & Co. Kg, Northeim, Germany). In the lower chambers, 750 μl conditioned medium from hAoSMCs subjected to scramble siRNA + IFN-γ or anti-RSAD2 siRNA + IFN-γ in the absence or presence of 10 nM SCH 546,738 was added. Complete SMC medium was used as negative control, and complete SMC medium supplemented with 80 ng/mL CXCL12 (R&D systems) was used as positive control and was added in the lower chambers to validate the assay’s reliability. The CD14^+^ cells were left to migrate for 2 h at 37 °C and 5% CO_2_. The inserts were discarded, and the CD14^+^ cells in the lower chambers were left to settle down for 15 min in the incubator prior to capturing five images from five predetermined spots (2.7mm^2^/spot) per well. The number of cells in five spots was determined by manual counting.

For migration of T-cells, untouched CD3^+^ cells were cultured in 24-well culture plates (Sarstedt Inc.) at a density of 1 × 10^6^ cells per ml of RPMI 1640 GlutaMAX™, supplemented with 0.5 μM 2-β-mercaptoethanol, 10 U/ml PEST, 10 mM HEPES, 1 mM sodium pyruvate and 1 × non-essential amino acids, 10% FBS (Life Technologies). As resting CD3^+^ T cells do not express CXCR3, they were incubated with 25 μl ImmunoCult™ Human CD3/CD28/CD2 T cell activator beads (STEMCELL Technologies) at 37 °C and 5% CO_2_ for 5 days with half of the medium getting replaced with fresh medium after 72 h. Activated CD3^+^ cells were stained and processed according to the protocol for CD14^+^ cells, described above, except for some modifications. Here, 100 ng/ml human recombinant IL-2 was added to the cells prior to their migration and a combination of both CXCL12 and CXCL11 were used as positive control to reliably evaluate the migration of these cells in vitro.

### Immunohistochemistry and immunofluorescence staining of formalin-fixed paraffin-embedded tissues

Atherosclerotic biopsies from patients included in the Atherosclerosis- Lipids and Inflammation in Peripherial Arterial disease and Carotid Artery disease (ALIPACA) study were used. Patients undergoing carotid and femoral endarterectomy at Örebro University hospital, Central hospital in Karlstad and Västmanlands hospital in Västerås, Sweden, were included. Carotid atherosclerotic biopsies removed during surgery were fixed in formalin and embedded in paraffin. Formalin-fixed paraffin-embedded plaques were sectioned at 5 µm using MICROM HM 355S (Thermo Scientific). The folds were removed by water bath at 37 °C and the sections were dried at 60 °C for 30 min on an HP-3 hot plate (Histolab Products AB, Gothenburg, Sweden) prior to clearing and rehydrating the tissue sections in decreasing concentrations of ethanol and finally deionized water for 5 min. The tissue slides were then placed in 1 × Diva decloaker solution (Biocare Medical, Concord, California, USA) for heat-induced epitope retrieval at 95 °C and 20 min in a pressure cooker. The Diva buffer was cooled down to RT and eliminated under running deionized water and the tissue sections were permeabilized with TBS containing 0.1% Triton-X100 (Sigma-Aldrich) for 10 min at RT. For immunohistochemistry, the background peroxidase activity was blocked by incubating the sections with peroxidazed-1 solution (Biocare Medical) for 5 min prior to blocking non-specific background staining with Background sniper (Biocare Medical) for 15 min at RT. The sections were stained with rabbit anti-human RSAD2 (1:50, catnr. HPA041160, lot. A96738, Atlas antibodies), mouse anti-human von Willebrand factor (vWF) clone F8/86 (1:100, catnr. M0616, Agilent, Santa Clara, California USA), mouse anti-human smooth muscle actin (SMA) clone 1A4 (1:500, catnr. M0851, Agilent, Santa Clara, California, USA), mouse anti-human CD68 (1:50, catnr. NCL-L-CD68, Leica, Weitzlar, Germany) for 1 h at RT in a moist chamber. RSAD2 was stained in carotid arteries from at least three patients.

For immunohistochemistry, the sections were incubated with mouse probe (where mouse antibody was used) for 15 min prior to incubation with HRP-polymer for 30 min at RT. 3, 3’-diaminobenzidine substrate solutions was added to the tissue sections and incubated for up to 5 min at RT prior to washing and dehydration by placing the slides in an increasing concentration of ethanol and finally xylene. The slides were mounted with Pertex mounting medium (Histolab Products AB), left to dry and scanned with Panoramic 250 (3DHISTECH Kft, Budapest, Hungary).

Immunofluorescence staining was performed similarly, except that the tissues were blocked with PBS containing 0.2 M lysine and 2% BSA instead of peroxidazed and background sniper for 1 h prior to incubation with the cocktail of primary antibodies (same concentrations as mentioned above) for another 1 h at RT. The sections were thoroughly washed and incubated with the cocktail of secondary antibodies consisting of goat anti-mouse Cy3-conjugated IgG (1:1000, catnr. A10521, Thermo Scientific) and goat anti-rabbit Cy5-conjugated IgG (1:200, catnr A10523, Thermo Scientific) for 2 h at RT in a moist chamber. The sections were washed and incubated with pre-mixed VECTASHIELD TrueView Autofluorescence Quencher (Vector Laboratories, Burlingame, California, USA) for 3 min. The slides were mounted with VECTASHIELD Plus Antifade Mounting Medium with DAPI (Vector Laboratories, Burlingame, California, USA) and left to dry in dark before scanning with Panoramic MIDI (3DHISTECH Kft, Budapest, Hungary).

### Human carotid atheroma gene expression data

Gene expression data from human atherosclerotic tissues were obtained from an existing dataset (GSE43292) within the Gene Expression Omnibus database. The detailed design and patients’ characteristics of this study is described by Ayari and Bricca^27^. In brief, plaques from 32 hypertensive patients, undergoing carotid endarterectomy at Lyon University Hospital, were collected and subsequently dissected into macroscopically intact (stage I-II) or atherosclerotic tissues (stage IV or higher) according to the Stary’s classification atherosclerotic tissues^28^. mRNAs from these sections were extracted and quantified by Affymetrix Human GeneChip Gene 1.0 ST Arrays. The data was analyzed using GEO2R web tool. The distribution of values across the samples were normalized and cross-comparable.

#### Statistical analysis

For assessing statistical differences, one-way ANOVA followed by Dunnett’s test was used for comparison of multiple groups with control group. One-way ANOVA followed by Bonferroni’s multiple comparison test was used for comparison of two groups in the presence of more than two groups. The data represents at least three independent experiments, unless otherwise indicated. For the human atheroma gene expression data, obtained from Gene Expression Omnibus with accession: GSE43292, the differences in gene expression were evaluated with paired t-test and Benjamini and Hochberg corrections were employed to obtain adjusted *p* values. Pearson’s correlation was used to evaluate the correlation between genes in lesions. *p* values smaller than 0.05 were considered statistically significant. All the data were analyzed in Prism version 9 (GraphPad Software Inc., San Diego, California, USA).

#### Ethics approval

The use of human blood and plaques for this study was approved by the Regional Ethical Review board in Uppsala, Sweden (Dnr: 215/543 and Dnr: 2018/207, respectively). Written informed consents were obtained from all participants and the study was in accordance with the declaration of Helsinki.

### Supplementary Information

Supplementary Information.

## Data Availability

The human carotid plaque data is accessible at National Center for Biotechnology Information’s webpage under the accession number GSE43292, https://www.ncbi.nlm.nih.gov/geo/geo2r/?acc=GSE43292.
