# Supplementary material for: RSAD2 is abundant in atherosclerotic plaques and promotes interferon-induced CXCR3-chemokines in human smooth muscle cells
Source: Sci Rep. 2024 Apr 8;14:8196. doi: 10.1038/s41598-024-58592-9 (PMC11001978; doi:10.1038/s41598-024-58592-9)
Supplement: Supplementary file 1 — Supplementary Information. [file 41598_2024_58592_MOESM1_ESM.pdf]

Supplementary material

RSAD2 IS ABUNDANT IN ATHEROSCLEROTIC PLAQUE AND PROMOTES INTERFERON-INDUCED CXCR3-CHEMOKINES IN HUMAN SMOOTH MUSCLE CELLS

Assim Hayderi*, Ashok K. Kumawat, Vladimir S. Shavva, Mats Dreifaldt, Birgitta Sigvant, Marcelo H. Petri, Björn Kragsterman, Peder S. Olofsson, Allan Sirsjö, Liza U. Ljungberg

*Correspondence to: assim.hayderi@hotmail.com

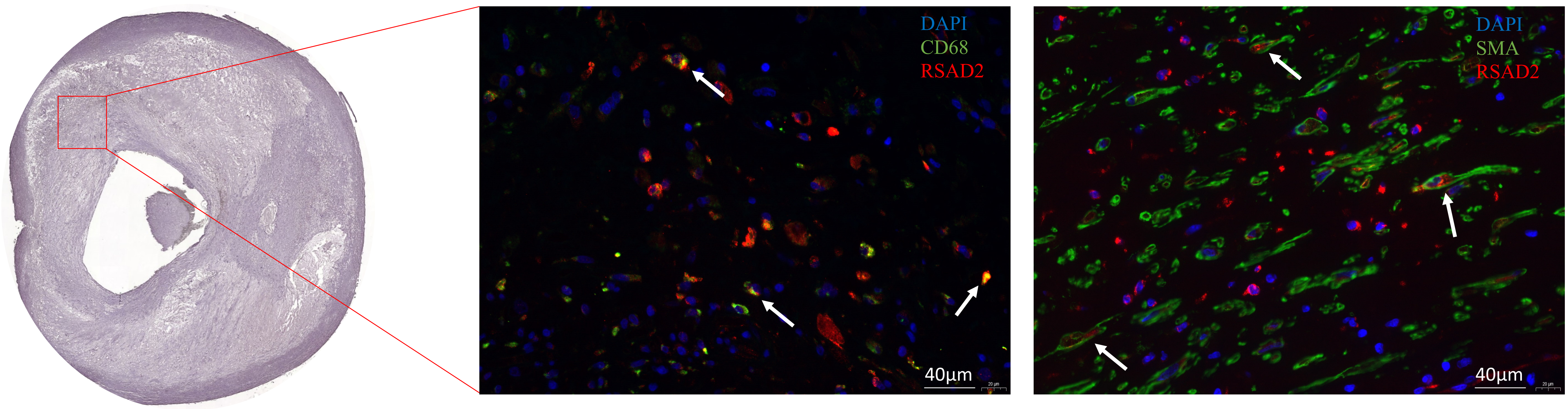

Fig. S1: RSAD2 is expressed in human carotid atherosclerotic lesions. Large field immunofluorescence microscopy images showing RSAD2 expression in the areas contianing macrophages and SMCs within human carotid atherosclerotic lesions.

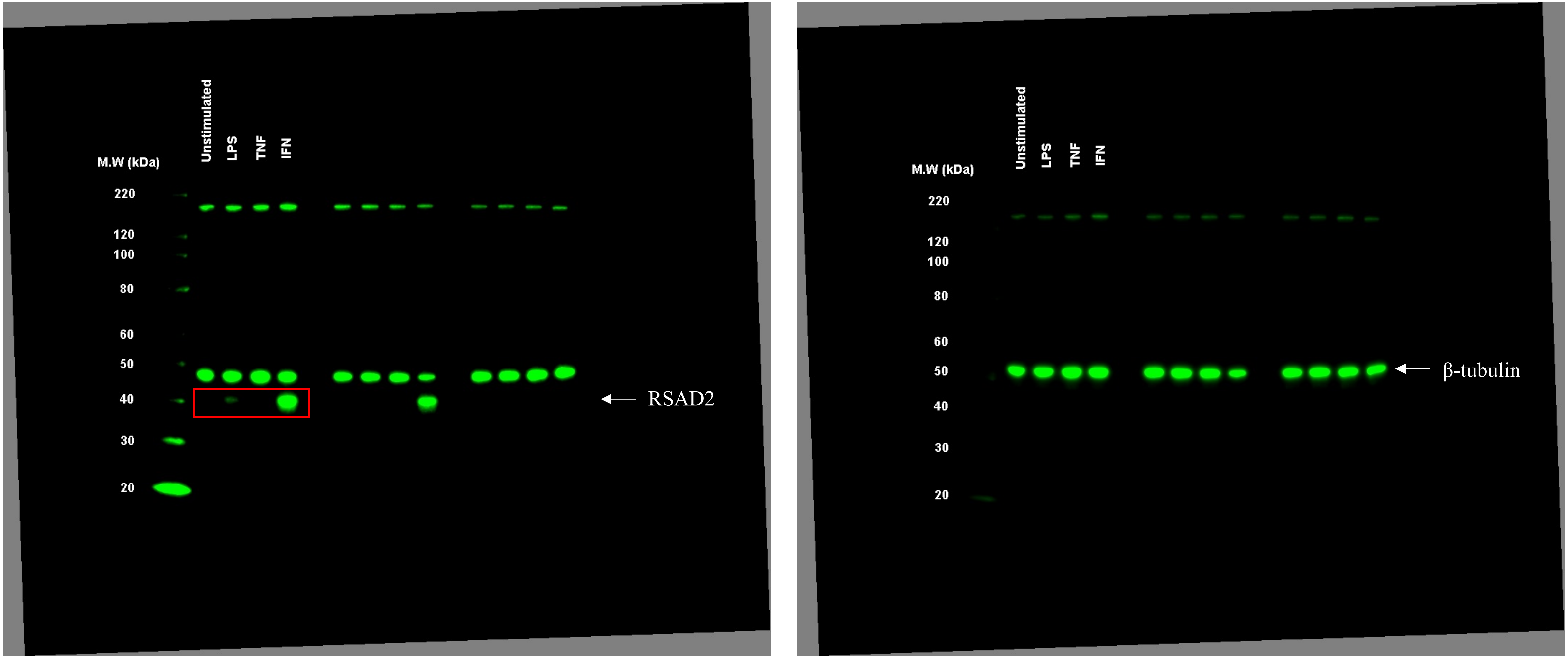

Fig. S2a: Full-length Western blot images representing figure 2c and showing RSAD2 and β-tubulin staining (experiment 1). Full-length Western blot images depicting the molecular size of markers (lane 1), RSAD2 staining (left), and β-tubulin staining (right) in cell lysates obtained from unstimulated cells (lane 2), cells stimulated with LPS (lane 3), TNF-α (lane 4) or IFN-γ (lane 5) for 48 hours (red box marks the region shown in the main text). These data are from experiment 1 and originate from the same gel/membrane. RSAD2 antibody (catnr. HPA041160, lot. A96738) was procured from Atlas Antibodies, with validation carried out for both Western Blotting and immunohistochemistry by Human Protein Atlas Project, while the β-tubulin antibody (clone AA2, catnr. 05-661, lot. 2913549) was acquired from Millipore.

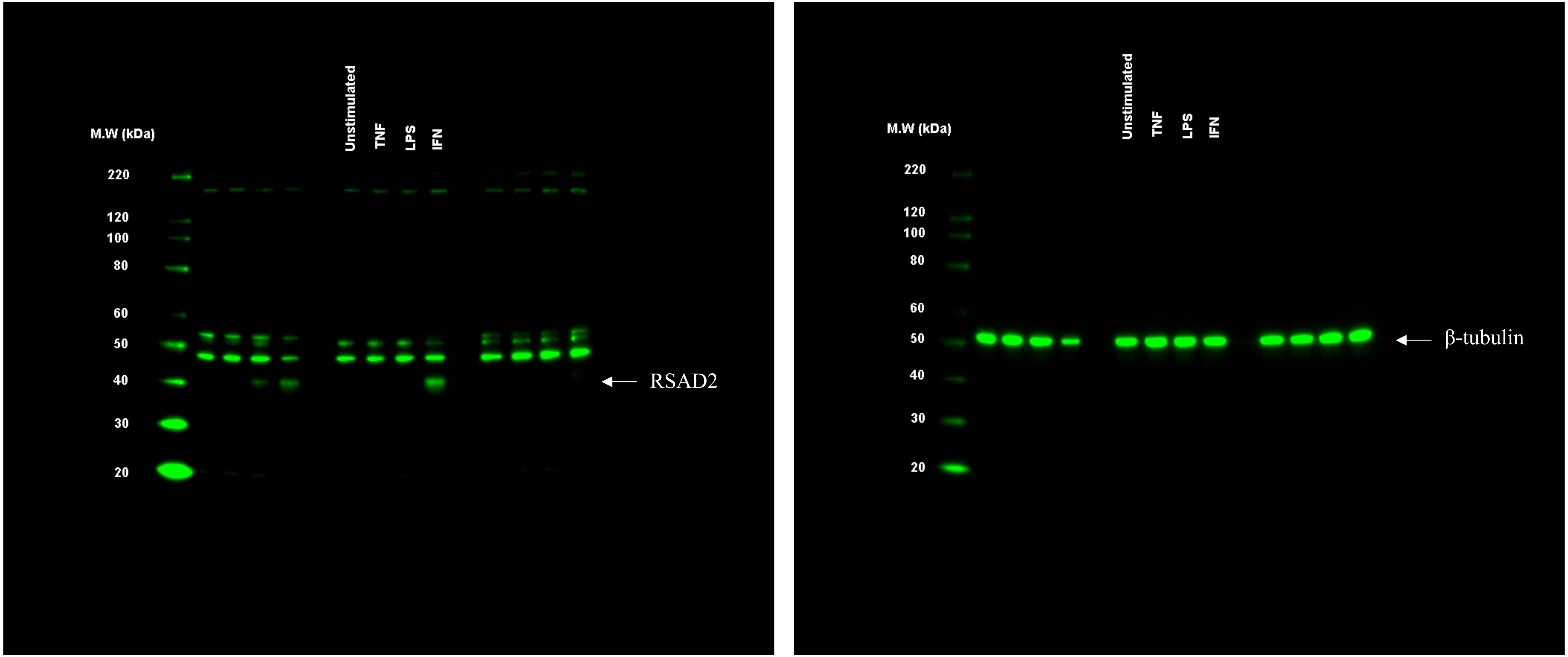

Fig. S2b: Full-length Western blot images representing figure 2c and showing RSAD2 and β-tubulin staining (experiment 2). Full-length Western blot images depicting the molecular size of markers (lane 1), RSAD2 staining (left), and β-tubulin staining (right) in cell lysates obtained from unstimulated cells (lane 6), cells stimulated with TNF-α (lane 7), LPS (lane 8) or IFN-γ (lane 9) for 48 hours. These data are from experiment 2 and originate from the same gel/membrane. RSAD2 antibody (catnr. HPA041160, lot. A96738) was procured from Atlas Antibodies, with validation carried out for both Western Blotting and immunohistochemistry by Human Protein Atlas Project, while the β-tubulin antibody (clone AA2, catnr. 05-661, lot. 2913549) was acquired from Millipore.

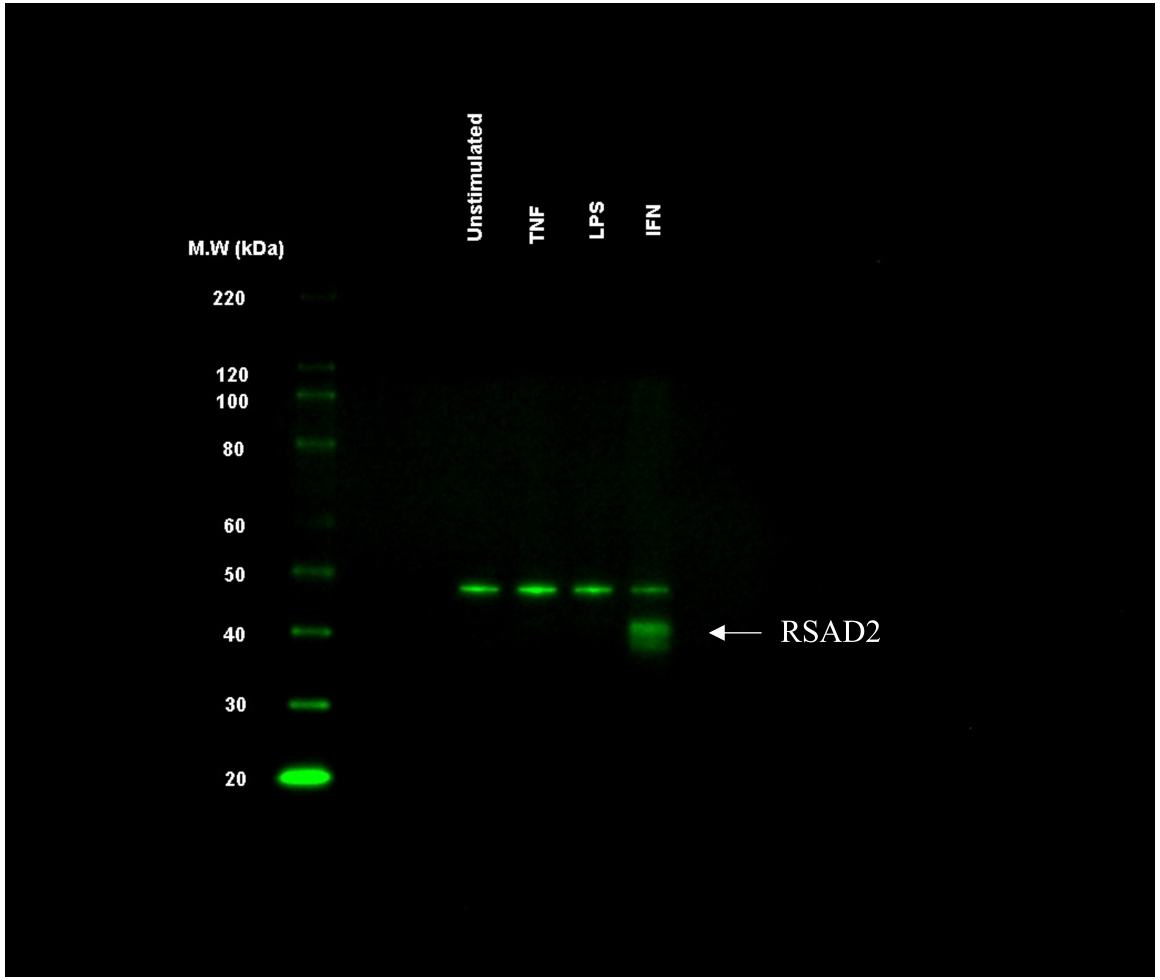

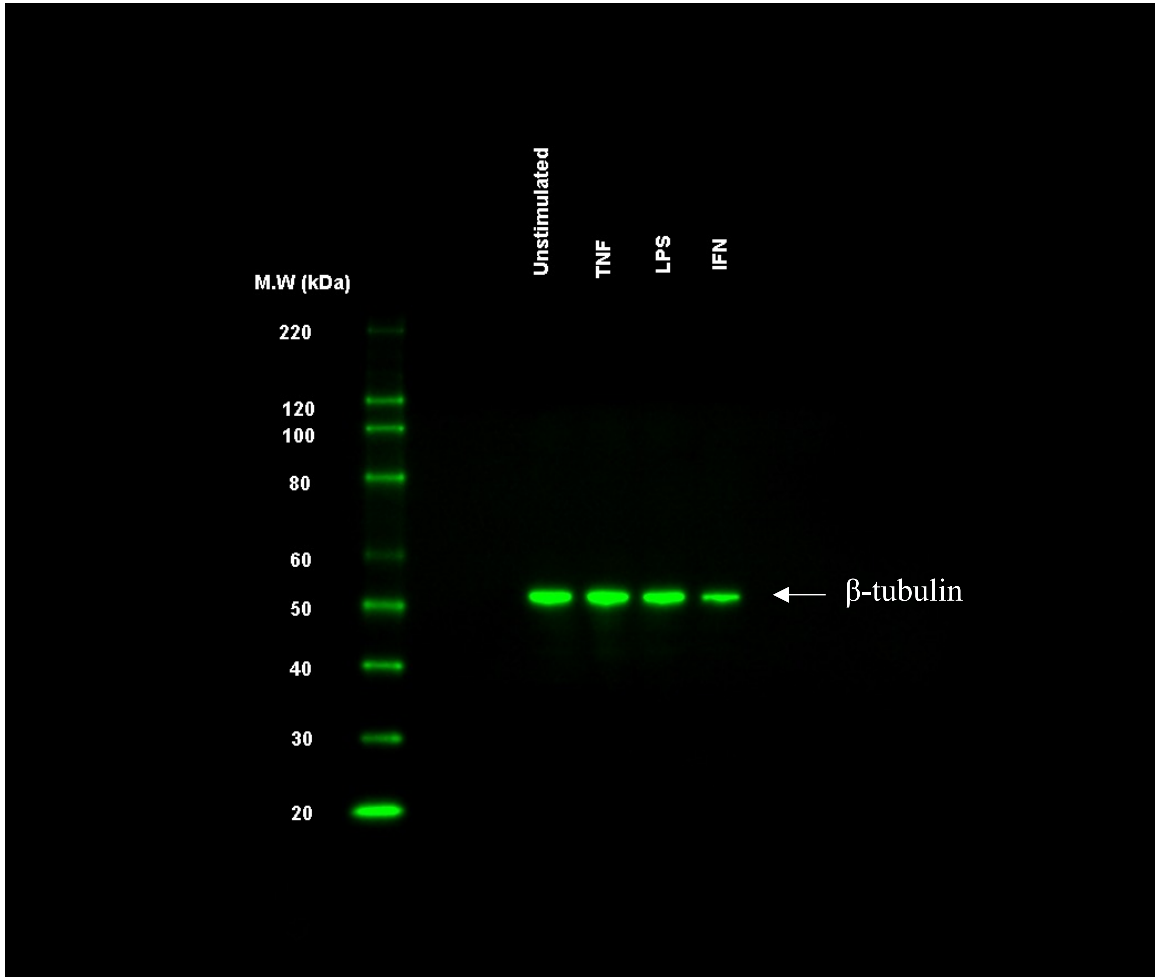

Fig. S2c: Full-length Western blot images representing figure 2c and showing RSAD2 and β-tubulin staining (experiment 3). Full-length Western blot images depicting the molecular size of markers (lane 1), RSAD2 staining (left), and β-tubulin staining (right) in cell lysates obtained from unstimulated cells (lane 2), cells stimulated with TNF-α (lane 3), LPS (lane 4) or IFN-γ (lane 5) for 48 hours. These data are from experiment 3 and originate from the same gel/membrane. RSAD2 antibody (catnr. HPA041160, lot. A96738) was procured from Atlas Antibodies, with validation carried out for both Western Blotting and immunohistochemistry by Human Protein Atlas Project, while the β-tubulin antibody (clone AA2, catnr. 05-661, lot. 2913549) was acquired from Millipore.

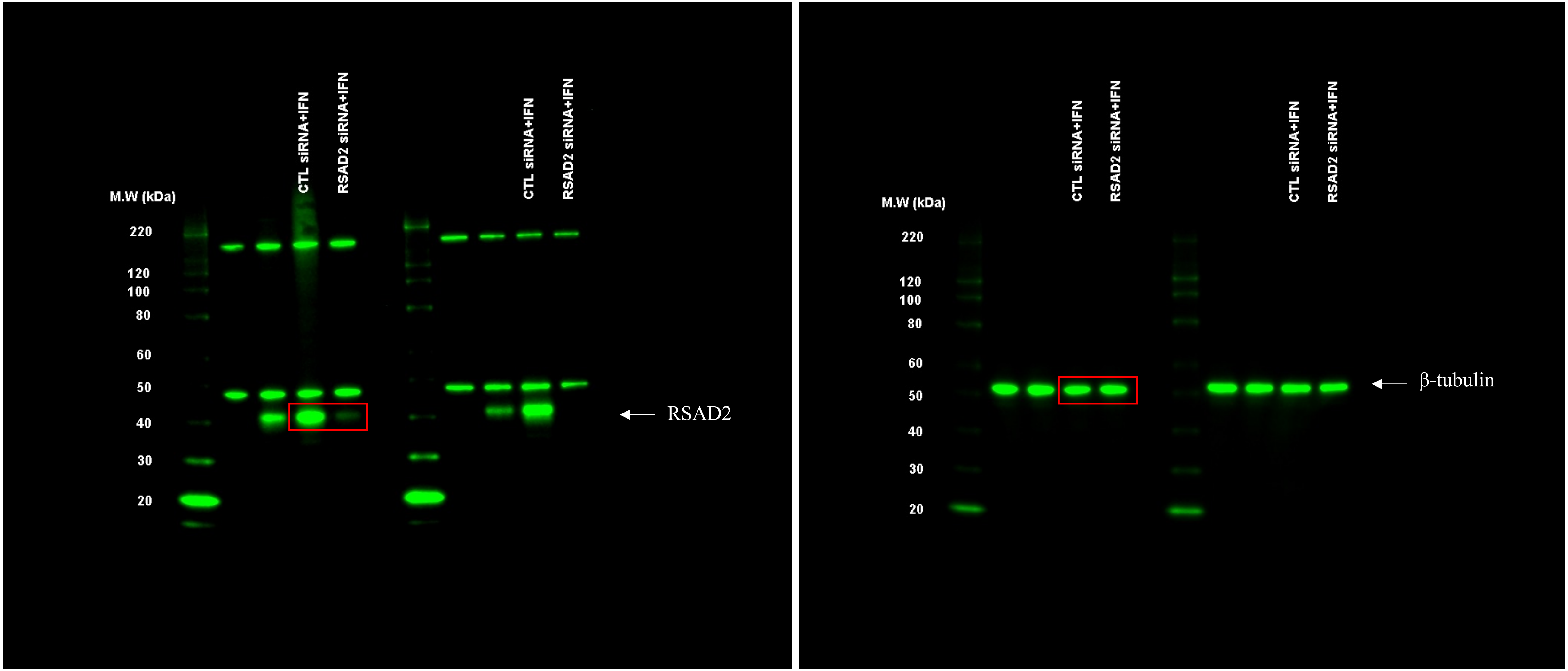

Fig. S3a: Full-length Western blot images representing figure 2f and showing RSAD2 and β-tubulin staining (experiment 1 and 2). Full-length Western blot images depicting the molecular size of markers (lane 1 and 6), RSAD2 staining (left), and β-tubulin staining (right) in cell lysates obtained from cells treated with control siRNA+IFN-γ (lane 4 and 9, red box marks the region shown in the main text) and RSAD2 siRNA+IFN-γ (lane 5 and 10) for 48 hours. These data are from experiment 1 and 2 and originate from the same gel/membrane. RSAD2 antibody (catnr. HPA041160, lot. A96738) was procured from Atlas Antibodies, with validation carried out for both Western Blotting and immunohistochemistry by Human Protein Atlas Project, while the β-tubulin antibody (clone AA2, catnr. 05-661, lot. 2913549) was acquired from Millipore.

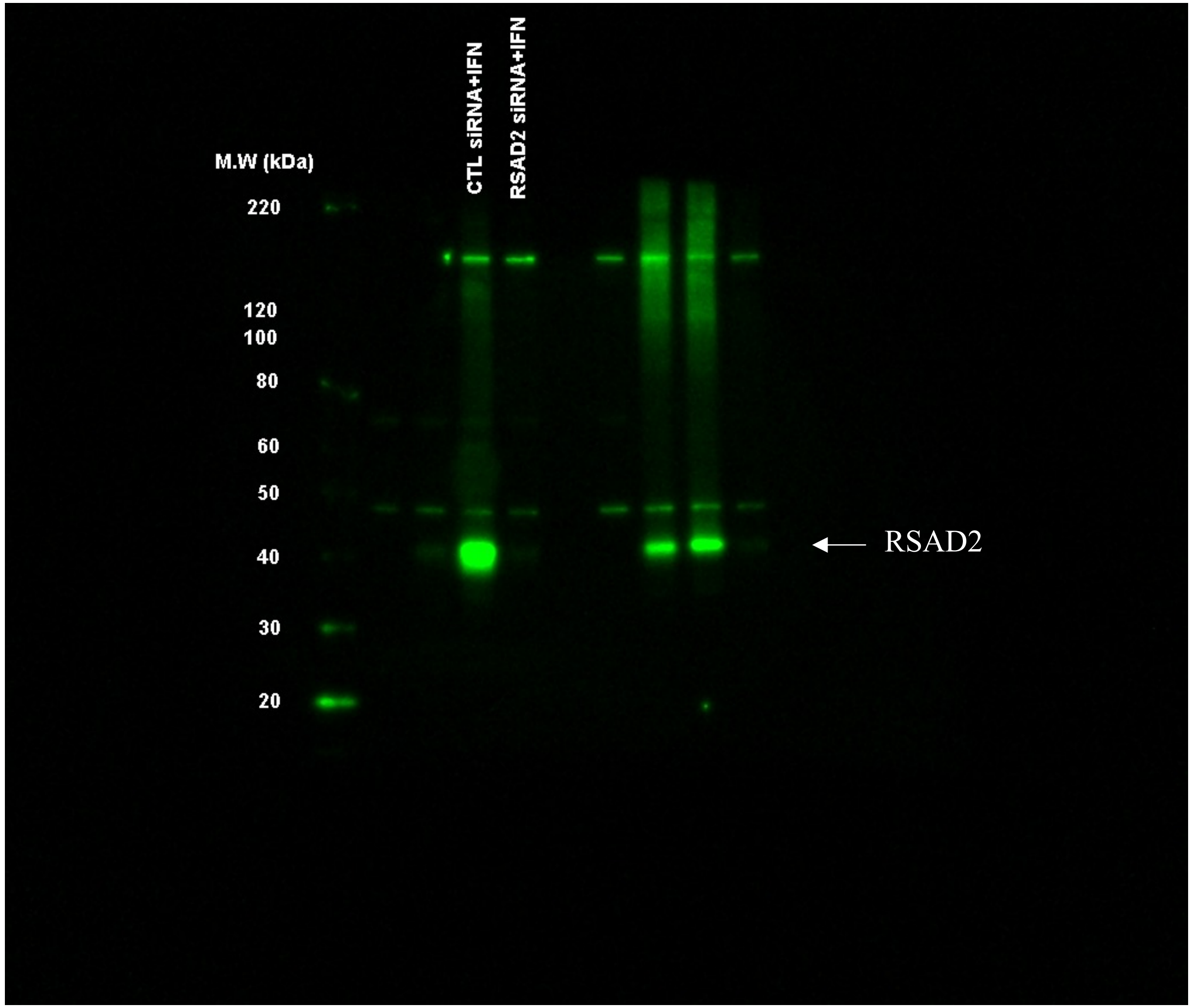

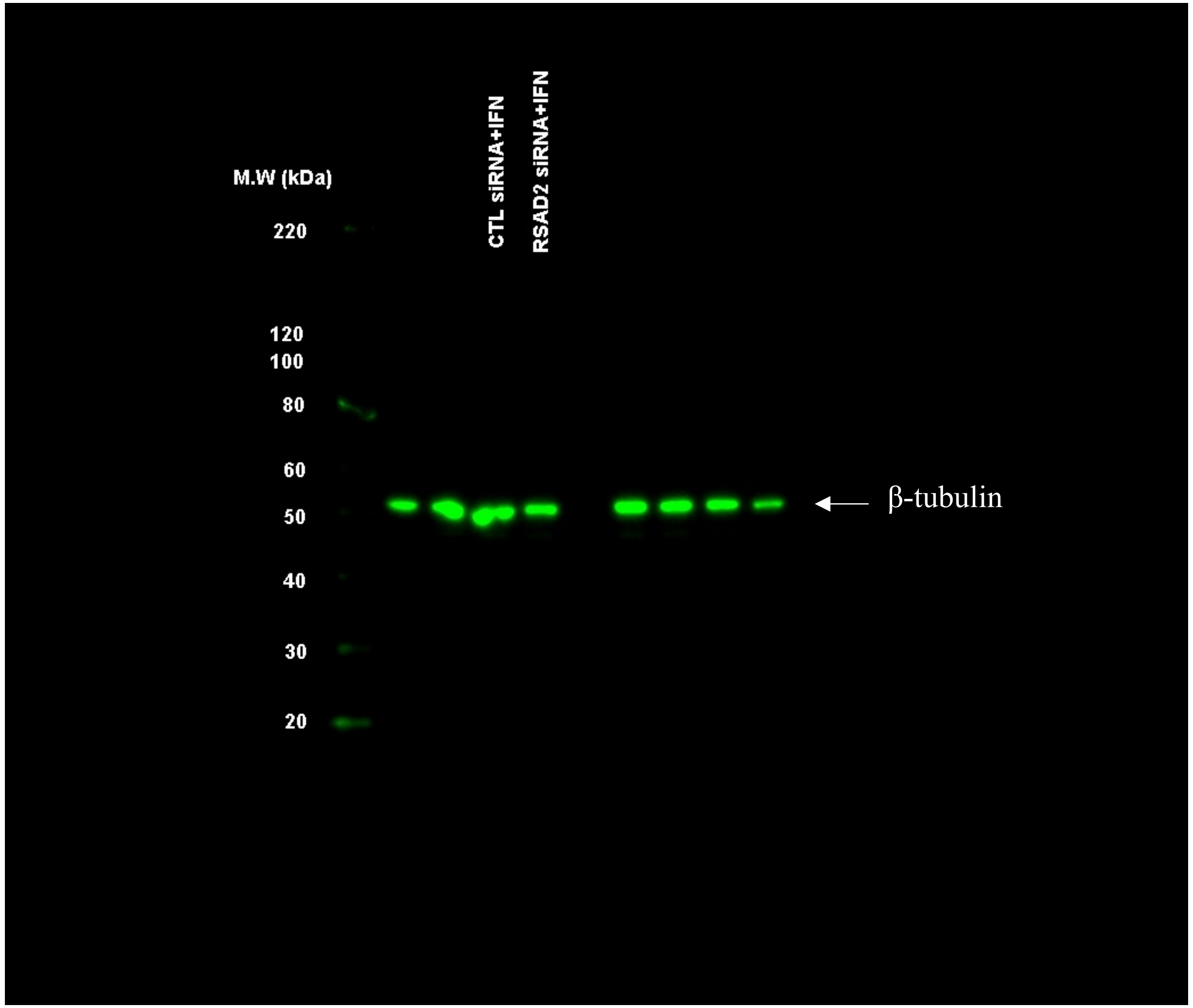

Fig. S3b: Full-length Western blot images representing figure 2f and showing RSAD2 and β-tubulin staining (experiment 3). Full-length Western blot images depicting the molecular size of markers (lane 1), RSAD2 staining (left), and β-tubulin staining (right) in cell lysates obtained from cells treated with control siRNA+IFN-γ (lane 4) and RSAD2 siRNA+IFN-γ (lane 5) for 48 hours. These data are from experiment 3 and originate from the same gel/membrane. RSAD2 antibody (catnr. HPA041160, lot. A96738) was procured from Atlas Antibodies, with validation carried out for both Western Blotting and immunohistochemistry by Human Protein Atlas Project, while the β-tubulin antibody (clone AA2, catnr. 05-661, lot. 2913549) was acquired from Millipore.

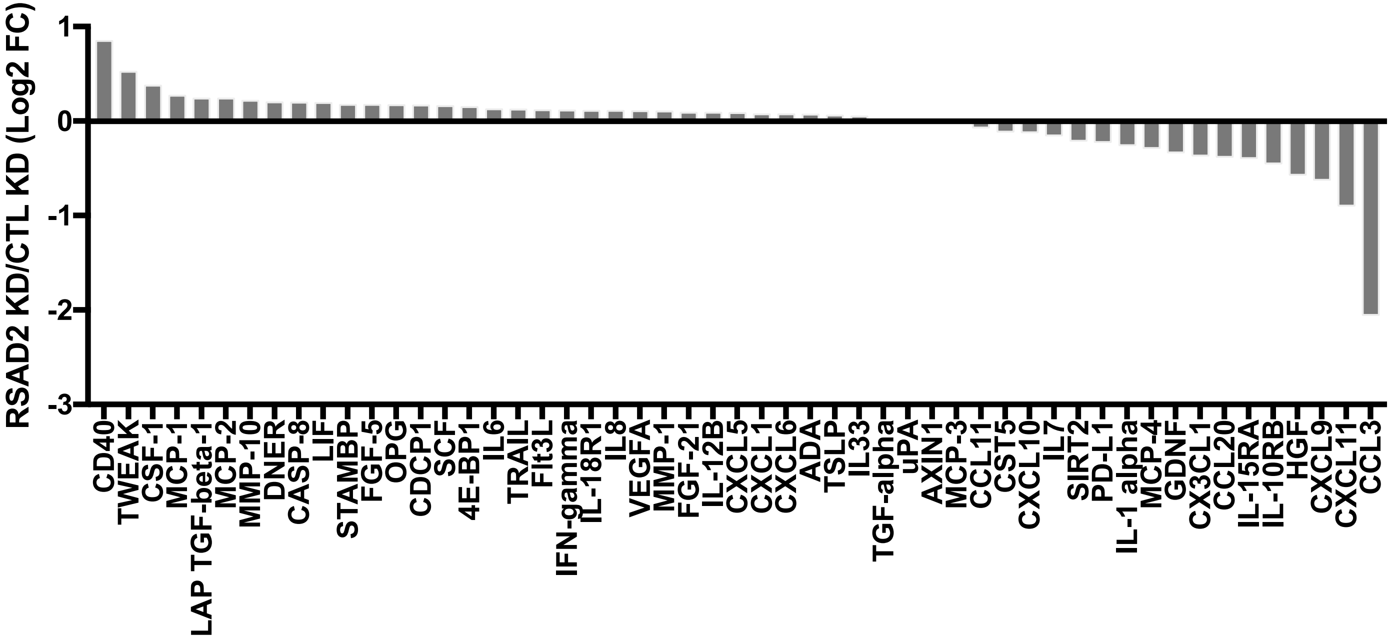

Fig. S4: The protein expression of CXCL9, CXCL10 and CXCL11 tends to be lower in IFN-γ-stimulated hAoSMCs that were transfected with RSAD2-specific siRNA. OLINK proteomics data (n=1) showing the log2 fold change of the detected proteins in inflammation panel from supernatant of IFN-γ-stimulated cells that were transfected with RSAD2 or CTL siRNA.

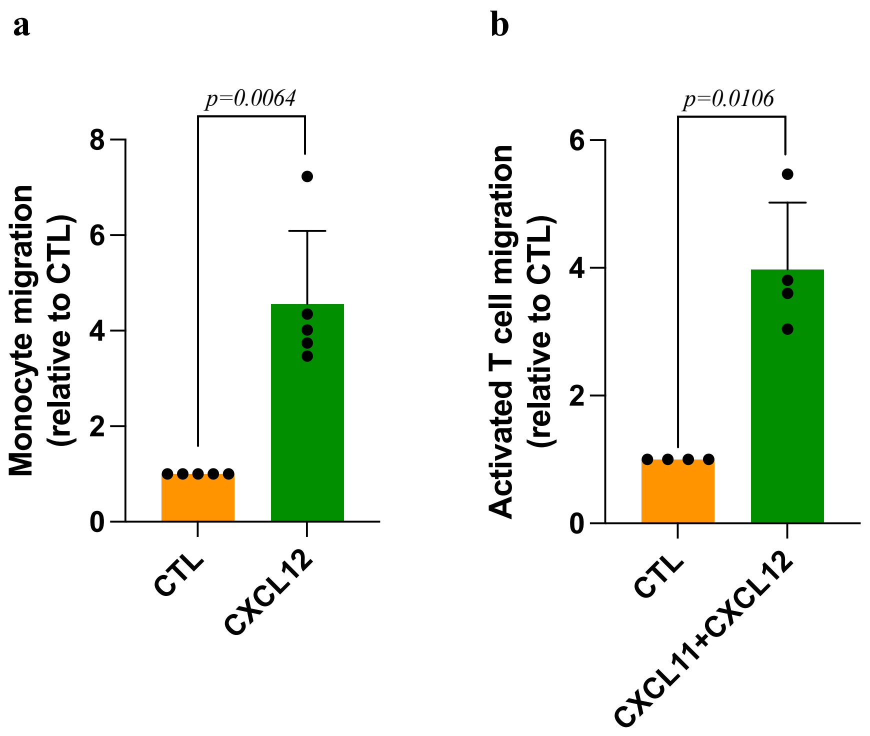

Fig. S5: Monocytes and activated T cells migrate more towards medium containing CXCL12 or CXCL12 in combination with CXCL11 in transwells. (A) Data showing higher abundance of (a) monocytes and (b) activated T cells migrating towards medium supplemented with CXCL12 or CXCL12 and CXCL11, respectively, for two hours, validating the reliability of the transwell assay. P-value <0.05 is considered statistically significant.

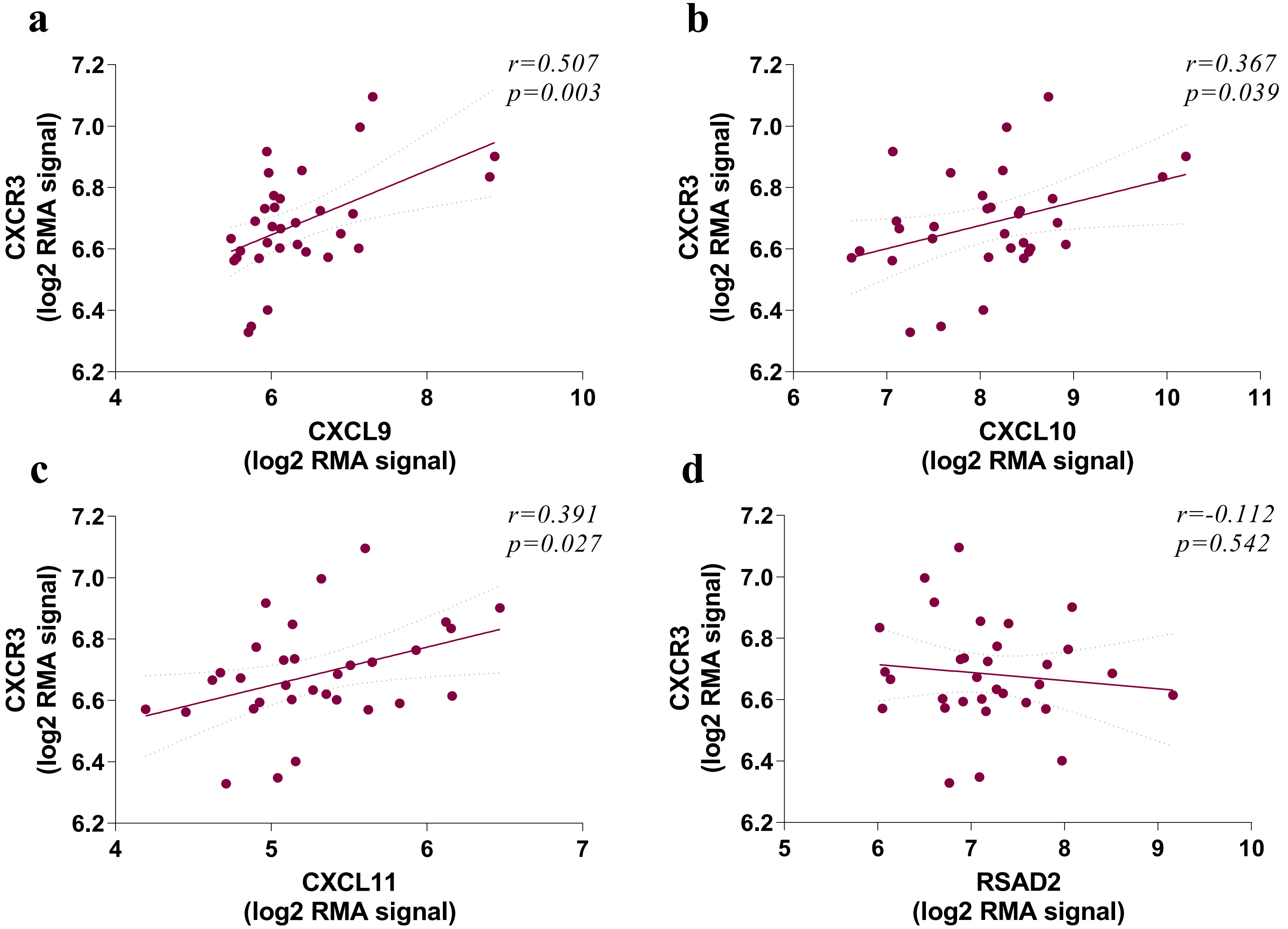

Fig. S6: RSAD2 shows no correlation with CXCR3 although CXCL9, CXCL10 and CXCL11 correlate with CXCR3 in atherosclerotic lesions. Pearson's correlation of CXCR3 with (A) CXCL9, (B) CXCL10, (C) CXCL11 and (D) RSAD2 in human carotid atherosclerotic lesions (n=32). Pearson's correlation coefficient, r, and p-values are indicated in the upper right corner. P-value<0.05 is considered statistically significant.

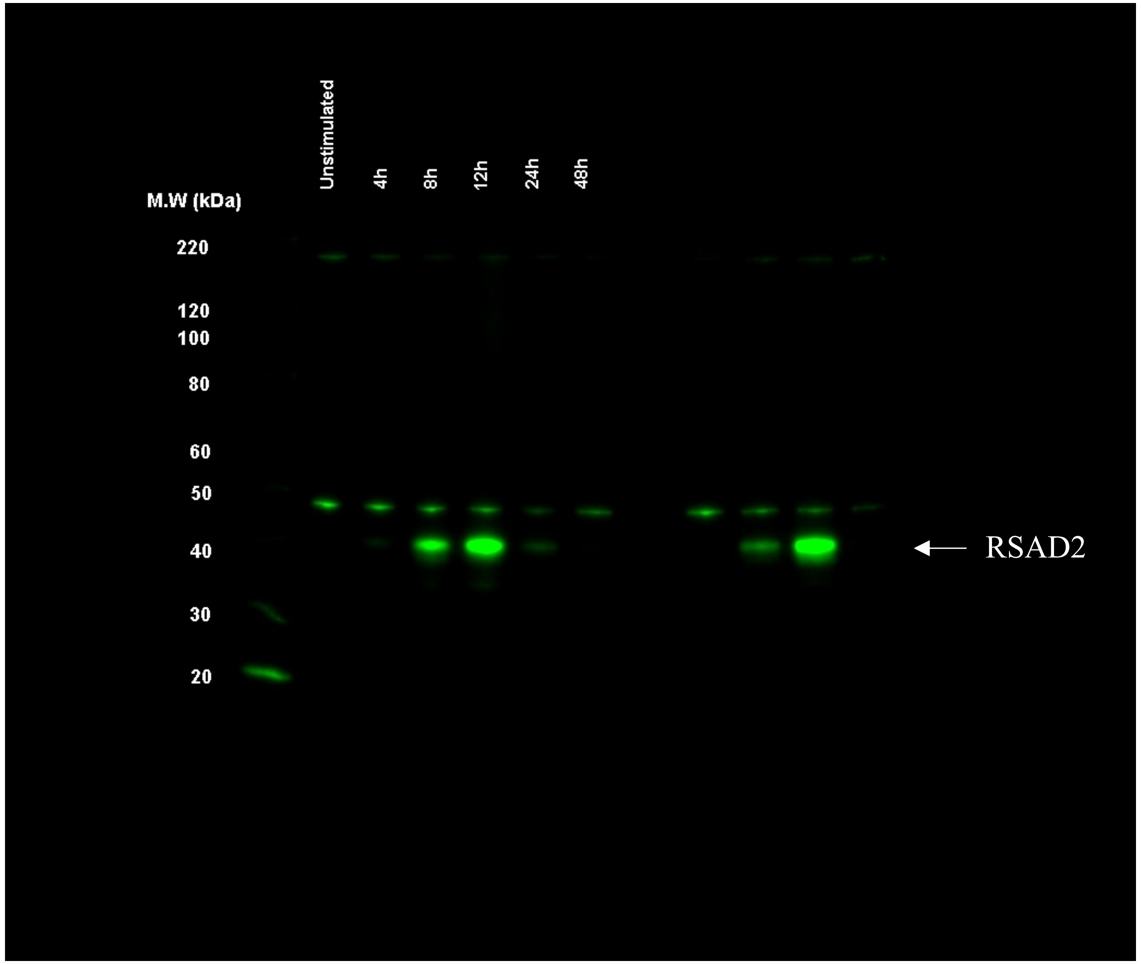

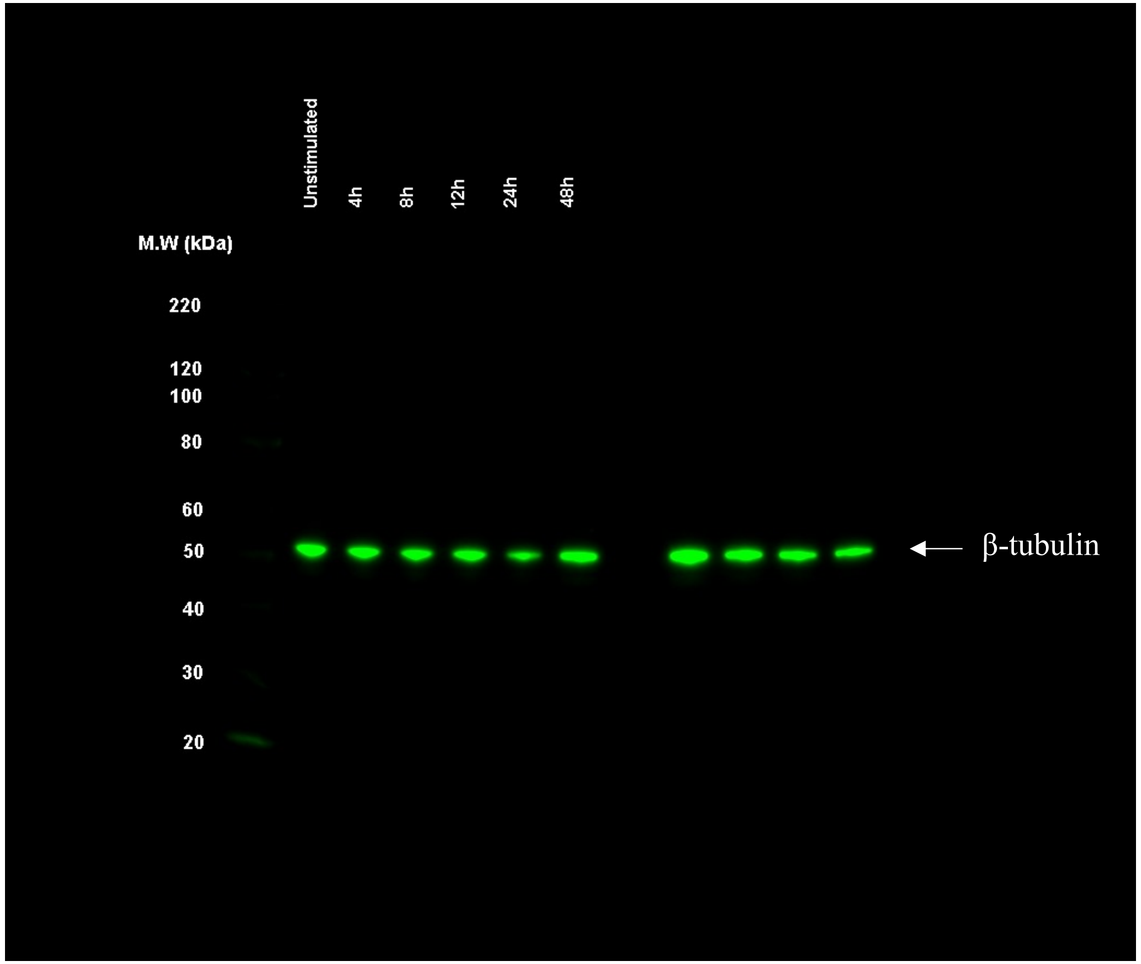

Fig. S7: Full-length Western blot images illustrating rapid induction of RSAD2 by LPS in hAoSMCs. Full-length Western blot images depicting the molecular size of markers (lane 1), RSAD2 (left) and β-tubulin (right) protein expression in lysates from hAoSMCs treated with LPS (100ng/ml) for 4-48 hours. These data originate from the same gel/membrane. RSAD2 antibody (catnr. HPA041160, lot. A96738) was procured from Atlas Antibodies, with validation carried out for both Western Blotting and immunohistochemistry by Human Protein Atlas Project, while the β-tubulin antibody (clone AA2, catnr. 05-661, lot. 2913549) was acquired from Millipore.

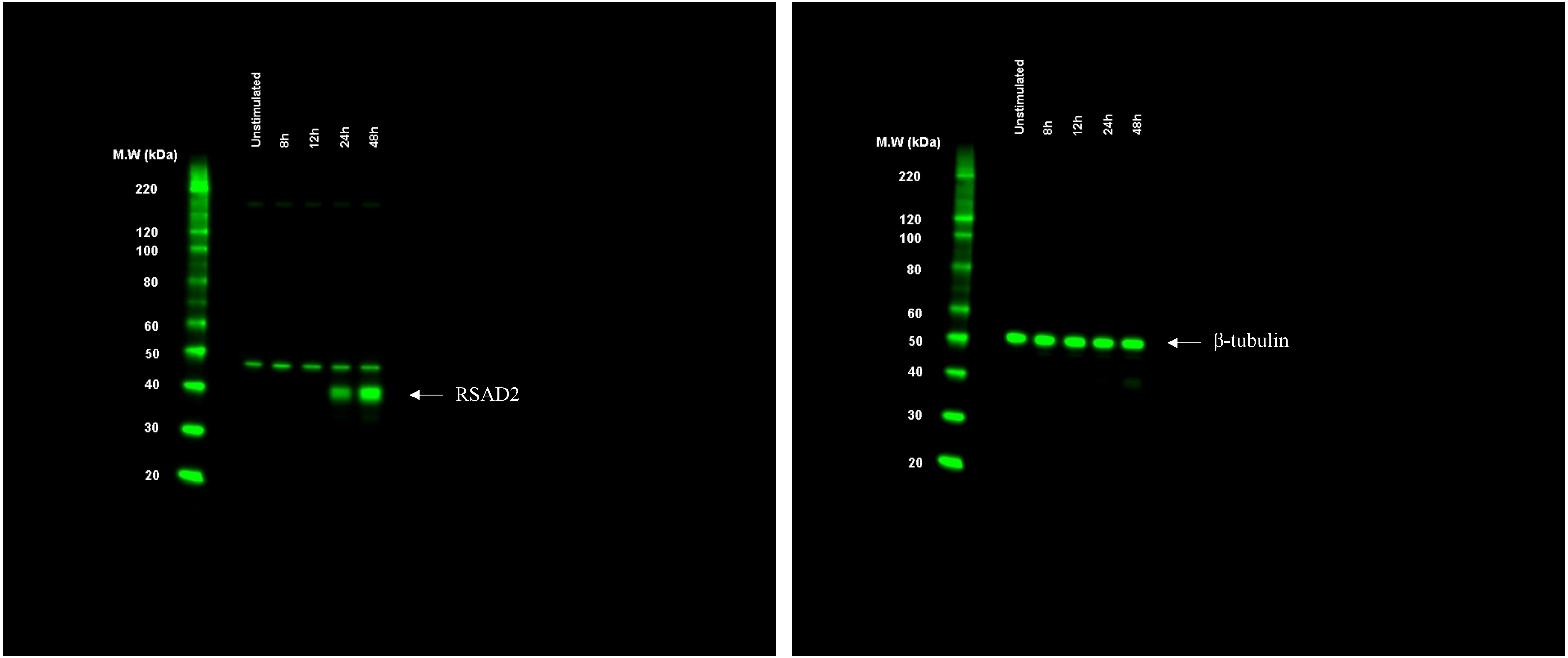

Fig. S8: Full-length Western blot images illustrating induction of RSAD2 by IFN-γ in hAoSMCs over 48 hours. Full-length Western blot images depicting the molecular size of markers (lane 1), RSAD2 (left) and β-tubulin (right) protein expression in lysates from hAoSMCs treated with IFN-γ (5ng/ml) for 8-48 hours. These data originate from the same gel/membrane. RSAD2 antibody (catnr. HPA041160, lot. A96738) was procured from Atlas Antibodies, with validation carried out for both Western Blotting and immunohistochemistry by Human Protein Atlas Project, while the β-tubulin antibody (clone AA2, catnr. 05-661, lot. 2913549) was acquired from Millipore.

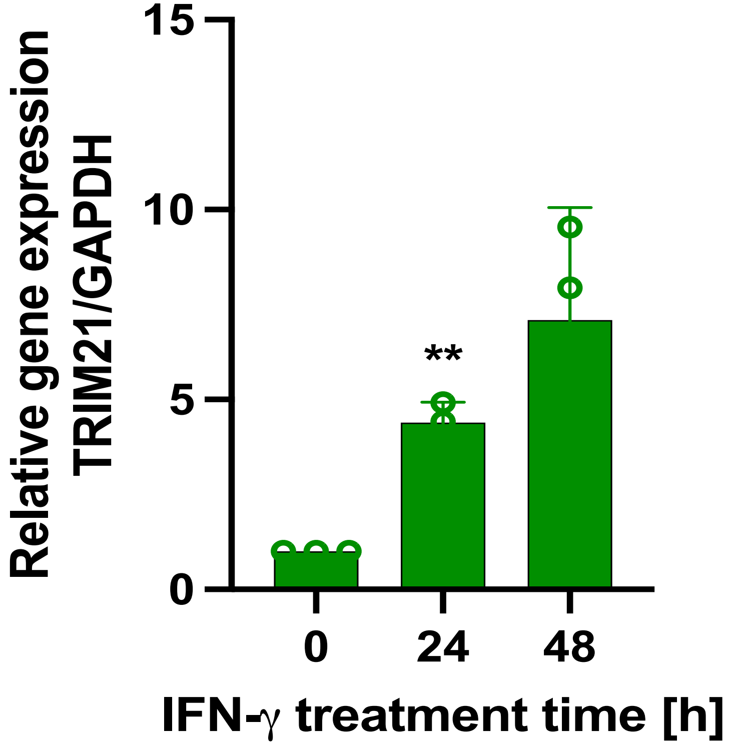

Fig. S9: Interferon-stimulated gene (ISG) induced by IFN-*γ* in hAoSMCs. Relative gene expression of TRIM21 after stimulation of hAoSMCs with IFN-*γ* for 24 and 48 hours. The data are presented as mean±SD of three independent experiments. One-sample test was used to determine statistical significance.

Table S1: RSAD2-correlating genes in human carotid lesions. Left table (orange) shows the list of top twenty genes that positively correlates with RSAD2 and the right table (green) contains the list of top twenty genes that negatively correlates with RSAD2 in human carotid atherosclerotic lesions. Log2 fold change and adjusted p-values are also indicated.

Gene symbol	Corr. Coefficient	Log2 FC	Adjusted p-value
IFIT1	0,949	0,368	0,076
IFI44L	0,931	0,888	0,001
USP41	0,923	0,454	0,001
IFIT3	0,922	0,567	0,000
IFIT2	0,910	0,331	0,026
MX1	0,908	0,181	0,004
HERC6	0,881	0,3	0,033
HERC5	0,875	-0,009	0,970
IFI44	0,854	0,906	0,000
OAS2	0,837	0,823	0,000
IFI6	0,808	0,568	0,001
EIF2AK2	0,805	0,051	0,654
CMPK2	0,799	0,276	0,001
IFIH1	0,799	0,399	0,000
XAF1	0,783	0,58	0,000
MX2	0,772	0,984	0,000
EPSTI1	0,769	0,858	0,000
DDX58	0,755	0,183	0,117
SAMD9L	0,749	0,517	0,000
CALCR	0,726	0,107	0,511

Gene symbol	Corr. Coefficient.	Log2 FC	Adjusted p-value
BMP1	-0,661	0,061	0,65
LOXL2	-0,615	0,196	0,22
ZDHHC16	-0,613	0,076	0,16
CRTAC1	-0,611	0,326	0,15
RBM14	-0,608	0,089	0,27
ZSCAN22	-0,606	0,052	0,43
FUT11	-0,605	-0,016	0,86
DNM1P46	-0,599	0,087	0,46
SH3PXD2B	-0,596	0,149	0,22
SEC14L2	-0,593	0,029	0,84
MMP2	-0,582	0,1	0,45
BCL6	-0,581	0,061	0,62
PPARA	-0,581	-0,018	0,88
COL3A1	-0,572	0,037	0,86
GFPT2	-0,567	0,377	0,026
EMILIN1	-0,567	-0,134	0,271
COL6A3	-0,566	0,33	0,069
AATF	-0,563	-0,011	0,89
SNED1	-0,562	-0,094	0,55
MOCOS	-0,560	0,085	0,28
